# Isolation and characterization of HepP: a virulence-related *Pseudomonas aeruginosa* heparinase

**DOI:** 10.1186/s12866-017-1141-0

**Published:** 2017-12-16

**Authors:** Nyaradzo Dzvova, Jane A. Colmer-Hamood, John A. Griswold, Abdul N. Hamood

**Affiliations:** 1 Department of Immunology and Molecular Microbiology, Texas Tech University Health Sciences Center, 3601 4th St. Mail Stop 6591, Lubbock, TX 79430 USA; 2 Department of Medical Education, Texas Tech University Health Sciences Center, Lubbock, TX USA; 3 Department of Surgery, Texas Tech University Health Sciences Center, Lubbock, TX USA

**Keywords:** *Pseudomonas aeruginosa*, Heparinase, Virulence, Pellicle, Biofilm, *C. elegans* killing model

## Abstract

**Background:**

*Pseudomonas aeruginosa* is an opportunistic pathogen that causes serious infections in immunocompromised hosts including severely burned patients. In burn patients, *P. aeruginosa* infection often leads to septic shock and death. Despite numerous studies, the influence of severe thermal injuries on the pathogenesis of *P. aeruginosa* during systemic infection is not known. Through RNA-seq analysis, we recently showed that the growth of *P. aeruginosa* strain UCBPP-PA14 (PA14) in whole blood obtained from severely burned patients significantly altered the expression of the PA14 transcriptome when compared with its growth in blood from healthy volunteers. The expression of *PA14_23430* and the adjacent gene, *PA14_23420*, was enhanced by seven- to eightfold under these conditions.

**Results:**

Quantitative real-time PCR analysis confirmed the enhancement of expression of both *PA14_23420* and *PA14_23430* by growth of PA14 in blood from severely burned patients. Computer analysis revealed that *PA14_23430* (*hepP*) encodes a potential heparinase while *PA14_23420* (*zbdP*) codes for a putative zinc-binding dehydrogenase. This analysis further suggested that the two genes form an operon with *zbdP* first. Presence of the operon was confirmed by RT-PCR experiments.

We characterized *hepP* and its protein product HepP. *hepP* was cloned from PA14 by PCR and overexpressed in *E. coli*. The recombinant protein (rHepP) was purified using nickel column chromatography. Heparinase assays using commercially available heparinase as a positive control, revealed that rHepP exhibits heparinase activity. Mutation of *hepP* resulted in delay of pellicle formation at the air-liquid interface by PA14 under static growth conditions. Biofilm formation by PA14Δ*hepP* was also significantly reduced. In the *Caenorhabditis elegans* model of slow killing, mutation of *hepP* resulted in a significantly lower rate of killing than that of the parent strain PA14.

**Conclusions:**

Changes within the blood of severely burned patients significantly induced expression of *hepP* in PA14. The heparinase encoded by *hepP* is a potential virulence factor for PA14 as HepP influences pellicle formation as well as biofilm development by PA14 and the protein is required for full virulence in the *C. elegans* model of slow killing.

**Electronic supplementary material:**

The online version of this article (10.1186/s12866-017-1141-0) contains supplementary material, which is available to authorized users.

## Background

The gram-negative opportunistic pathogen *Pseudomonas aeruginosa* causes severe infection in immunocompromised hosts such as HIV-infected patients, individuals with cystic fibrosis, cancer patients, transplant patients, and severely burned patients [1, 2]. Damage caused during different *P. aeruginosa* infections is due to the production of numerous cell-associated and extracellular virulence factors [2–5]. The extracellular factors include proteases such as elastase, hemolysins such as phospholipase C, siderophores such as pyoverdine, and toxins such as exotoxin A. The cell-associated virulence factors include flagellum, alginate and pili [6, 7].

A severe burn destroys the skin barrier and reduces the expression of both local and systemic immune responses [8–11]. Additionally, substances that are produced by the injured cells within the burned wound impair local host immune responses [8, 11, 12]. Although burn wounds are initially sterile they are quickly colonized by bacterial pathogens including *P. aeruginosa* [13, 14]. *P. aeruginosa* grows within the wound and translocates to the blood stream causing bacteremia which is followed by sepsis, septic shock and multiorgan failure. Despite numerous studies, the influence of severe burn injury on the pathogenesis of *P. aeruginosa* during systemic infection is not completely understood. We recently followed a unique approach to address this issue. Instead of growing *P. aeruginosa* in a regular laboratory medium, we grew it in whole blood from either healthy volunteers or severely burned patients and conducted a comparative transcriptome analysis [15]. RNA-seq analysis showed that the growth of *P. aeruginosa* strain UCBPP-PA14 (PA14) in whole blood obtained from severely burned patients significantly altered the expression of the PA14 transcriptome when compared with its growth in blood from healthy volunteers [15]. Among genes not reported in the previous study whose expression was enhanced were *PA14_23430*, which codes for a putative heparinase, and the adjacent gene *PA14_23420*.

Heparin and heparan sulfate glycosaminoglycans (HSGAGs), which consist of linear chains of disaccharide units of *N*-acetylated D-glucosamine α (1–4) linked to glucuronic acids, are present on the surface of different mammalian cells and the extracellular matrices [16]. Enzymes that degrade HSGAGs by specifically cleaving the 1–4 glycosidic link in heparin and heparan sulfate are known as heparanase or heparinase [16]. Mammalian heparanases cleave at the reducing end of glucuronic acid by a hydrolytic mechanism whereas bacterial heparinases depolymerize heparin and heparan sulfate by β-elimination cleavage [16, 17]. Heparanases, which are important modulators of the extracellular matrices, are present in normal cells including endothelial cells, cytotrophoblasts, platelets, mast cells, neutrophils, macrophages, and lymphocytes [16, 18–21]. Heparanase expression is increased in certain human carcinomas and hematological malignancies [20, 21]. Heparanases also function in non-tumorous processes including, tissue morphogenesis, regeneration and repair during embryonic development and wound repair [20–23].

The best-characterized bacterial heparinases are the three enzymes produced by *Pedobacter heparinus*, (previously known as *Flavobacterium heparinum*) heparinase I, II, and III [16, 17, 22, 24–27]. No significant homology exists between either the DNA sequences of the genes that code for the three heparinases or the amino acid sequences of the proteins [28]. The three heparinases proved to be valuable tools in analyzing different structural, biochemical and physiological properties of heparin and heparan sulfate-like glycosaminoglycans [18, 22, 24, 26]. Additional uses of *P. heparinus* heparinases include heparinase I as a heparin antagonist [16, 29] and heparinases I, II, and III to reduce neovascularization during tumor progression through their ability to alter the action of fibroblast growth factor [16, 20, 21, 27]. Despite the proposed beneficial effects of the *P. heparinus* heparinases, it is not known if heparinases play a role in the pathogenesis of bacterial pathogens, specifically the ones that cause systemic infection.

In this study, we report the characterization of *PA14_23430*, or *hepP*, and its protein product HepP. *hepP* was cloned from PA14 by PCR and overexpressed in *E. coli*. The recombinant protein (rHepP) was purified using nickel column chromatography. The heparinase assay using commercially available heparinase revealed that rHepP exhibits heparinase activity. The deletion of *hepP* resulted in the delay of pellicle formation at the air-liquid interface by PA14 under static growth conditions. The biofilm formation by PA14Δ*hepP* was also significantly reduced. In the *Caenorhabditis elegans* model of slow killing, the deletion of *hepP* resulted in a significantly lower rate of killing than that of the parent strain PA14.

To our knowledge this is the first time a heparinase, or heparin and heparin sulfate degrading enzyme, has been characterized from *P. aeruginosa*. Due to its important role in PA14 virulence and as *P. aeruginosa* antibiotic resistant mutants emerge at an alarming rate, HepP represent a potential target for new antimicrobial agents.

## Methods

### Strains, plasmids and general growth conditions

Bacterial strains and plasmids used in this study are listed in Table 1. The *P. aeruginosa* strain UCBPP_PA14 (PA14), originally isolated from an infected wound, or its specific transposon mutants, PA14Δ*hepP* and PA14Δ*zbdP*, were used in all the experiments [30, 31]. The strains were routinely grown at 37 °C in Luria Bertani (LB) broth. Gentamicin was added to the growth medium at a concentration of 60 μg/mL for the PA14 mutants and carbenicillin was added at 100 μg/mL for *E. coli* strains carrying plasmids.

**Table 1:** Strains and plasmids used in this study

Strains or plasmids	Characteristics	Reference
*Pseudomonas aeruginosa*		
PA14	UCBPP-PA14; prototropic strain isolated from infected wound	[30, 31]
PA14Δ*hepP*	PA14/MrT7::*PA14_23430–*480; Gm^R^	[30]
PA14Δ*zbdP*	PA14/MrT7::*PA14_23420–*118; Gm^R^	[30]
*Escherichia coli*		
TOP10	F- *mcrA* Δ(*mrr-hsdRMS-mcrBC*) Φ80*lacZ*ΔM15 Δ*lac*Χ74 *recA*1 *araD*139 Δ(*ara-leu*)7697 *galU galK rpsL* (Str^R^) *endA*1 *nupG*	Invitrogen
OP50	Uracil auxotroph; feeder strain for *Caenorhabditis elegans*	[49]
*Caenorhabditis elegans*		
BW54	Temperature sensitive embryonic lethal strain; proliferates at 15 °C; sterile at 25 °C	*Caenorhabditis* Genetics Center
Plasmids		
pBAD/Thio-TOPO	pBR322-derived expression vector; cloned genes expressed from P_BAD_; amino terminus HP-thioredoxin; carboxy terminus V5 epitope and 6XHis; Cb^R^	Invitrogen
pND1	pBAD/Thio-TOPO/*hepP*; Cb^R^	This study

*Cb* ^*R*^ carbenicillin resistant, *Gm* ^*R*^ gentamincin resistant, *Str* ^*R*^ streptomycin resistant

### Real time quantitative PCR (qPCR) and reverse transcription PCR (RT-PCR)

Purified RNA samples obtained during a previous study in which we examined changes in the transcriptome of PA14 when grown in whole blood from severely burned patients compared with its growth in whole blood from healthy volunteers [15] were used in this study. The blood samples were obtained under an IRB-approved protocol in compliance with ethical practices [15].

Additional RNA samples were collected to examine the effect of the *hepP* mutation on the expression of specific virulence factors. We grew the strains in specific media, LB broth for the expression of quorum sensing genes and calcium-deficient chelexed trypticase soy broth dialysate medium for the expression of the type three secretion system (T3SS) genes [32], that are designed to maximally express those virulence genes. Briefly, PA14 cultures were grown and subcultured as described above. At the 8 h time point, cultures grown in the specific medium were mixed with twice the volume of the culture of RNAprotect Bacteria Reagent (Qiagen). After a 5 min incubation at room temperature, the cells were pelleted and stored at −80 °C. RNA was extracted using the RNeasy Mini Kit (Qiagen) according to the manufacturer’s recommendations. To ensure purity, the RNA solution was digested with the RNase-free DNase set (Qiagen), the RNA was purified from the DNase by the RNA cleanup protocol (Qiagen), and quantified using a NanoDrop Spectrophotometer (NanoDrop Technologies).

Samples with an *A*_260/280_ ratio of 1.8–2.2 were used to synthesize cDNA by means of the QuantiTect Reverse Transcription kit (Qiagen). Two hundred ng of cDNA was mixed with *Power*SYBR Green Master mix (Applied Biosystems) and 250 nM of specific primer (Table 2). The StepOnePlus Real-Time PCR System (Applied Biosystems) was then used to detect and quantify amplification of the PCR product. Three independent replicates for RNA samples were used for each experiment; additionally, each qPCR reaction was set up in triplicate. Normalization of the cDNA quantity in the different experiments was done using the 30S ribosomal subunit RNA (*rpsL*) as an internal standard. The software StepOnePlus version 2.2.2 was used to analyze gene expression. Negative control samples containing RNA as a template and positive control samples containing genomic DNA as a template were included for each experiment. RT-PCR was done as above but the cDNA products were analyzed on agarose gels. Primers used for all experiments involving PCR, RT-PCR or qPCR were synthesized by Integrated DNA Technologies and are listed in Table 2 and (Additional file 1: Table S1).

**Table 2:** Primers used in this study

Name	Sequence	Use
*hepP-*For1	GGACTGGCAAGCTGATAGGA	Analysis of *hepP* transcription Analysis of operon
*hepP-*Rev1	CGATACGCAAGGAAAGAGGA	Analysis of *hepP* transcription Analysis of operon
*zbdP-*For1	CGGGTGTTGGCTATTGATTT	Analysis of *zbdP* transcription
*zbdP-*Rev1	AACAATCCGTCCACGCTTAC	Analysis of *zbdP* transcription
*zbdP-*For2	CTTCTGAGGACAAGGCTTCG	Analysis of operon
*hepP-*Rev2	GATAAGGCTCCCAACCTTC	Analysis of operon
*zbdP-*For3	AGCCGACTATCGCTTGTGAA	Confirmation of *hepP* mutation
*hepP-*Rev3	GTGCTTTTCGAGCAATGGAG	Confirmation of *hepP* mutation
*hepP-*ATG	ATGGCACTTCAAAAACTCGTCCG	Cloning *hepP*
*hepP-*[-TGA]	ACTCGTATTTGCTGGGGTGGAATT	Cloning *hepP*
Trx Forward	TTCCTCGACGCTAACCTG	Confirmation of cloning
pBAD Reverse	GATTTAATCTGTATCAGG	Confirmation of cloning
*hepP-*For2	CGCTGGTGCAACAAGTAGAA	Analysis of *hepP* transcription
*hepP-*Rev4	GCGTGATACATCGGAGACAA	Analysis of *hepP* transcription

Primers were purchased from Integrated DNA Technologies

### Cloning of *hepP*

The DNA sequence of *hepP* was amplified with GoTaq Green Master Mix (Promega) using primers described in Table 2 and genomic DNA from strain PA14 as a template. The 1650-bp amplicon was cloned into pBAD/Thio-TOPO (Invitrogen) to create plasmid pND1. The construction of pND1 was confirmed by restriction enzyme digestion and sequencing analysis. Recombinant HepP (rHepP) expressed from pND1 has the HP-thioredoxin fusion for efficient translation at the N-terminus and a 6xHis fusion for purification at the C-terminus plus the V5 epitope for localization of the protein by Western blotting.

### Expression and purification of recombinant HepP (rHepP)

Plasmid pND1 was transformed into chemically competent OneShot TOP10 *Escherichia coli* (Invitrogen). TOP10/pND1 was grown in 50 mL LB broth at 37 °C with shaking, expression of *hepP* was induced with 0.02% L-arabinose, and the cultures incubated for an additional 4 h. To avoid denaturation of the protein during purification, rHepP was purified under native conditions using a nickel-nitrilotriacetic acid affinity column (Ni-NTA, Qiagen) according to the manufacturer’s protocol. Briefly, following induction, the cells were centrifuged and the pellet was suspended in lysis buffer (50 mM NaH_2_PO_4_, 300 mM NaCl, 10 mM imidazole at pH 8). The cell suspension was sonicated for 10 cycles of a 10 s burst with a 10 s cooling between bursts. Lysed cells were centrifuged at 10,000 x *g* for 20 min. The supernatant was applied to a column containing 50% Ni-NTA agarose slurry. The column was capped and the mixture was rotated for 60 min at 4 °C. The column was then uncapped and the flow-through was collected. The column was washed twice with wash buffer (50 mM NaH_2_PO_4_, 300 mM NaCl, 20 mM imidazole at pH 8) and rHepP was eluted with four applications of elution buffer (50 mM NaH_2_PO_4_, 300 mM NaCl, 250 mM imidazole at pH 8). The concentration of the purified protein was estimated using the Bradford assay as previously described [33]. The purified protein was analyzed using 10% SDS-PAGE [34, 35]. In addition, we confirmed the production of the recombinant protein by immunoblotting experiments using anti-V5 antibody (Invitrogen).

### Heparinase activity plate assay

The method is based on the ability of heparinase to cleave heparin and heparan sulfate [36]. Different concentrations of the samples to be tested were spotted on plates containing porcine intestinal heparin (1-mg/mL) (Sigma-Aldrich), 0.25 M sodium acetate, and 0.0025 M calcium acetate in 1.5% agarose at pH 7. The plates were incubated for 1 h at 37 °C and then 2% protamine sulfate solution (Sigma-Aldrich) was poured over the plates. The plates were incubated for 1–2 h at room temperature until a white precipitate formed on the uninoculated portions of the plate. The presence of clear zones indicate heparinase activity, with zones of increasing intensity at the areas where higher amounts of heparinase were added. Commercially available *P. heparinus* heparinase III (0.5–1-U) (Sigma-Aldrich) was used as a positive control. The protein elution buffer was used as a negative control.

### Growth curve analysis

The strains were grown overnight in LB broth and subcultured into fresh LB broth to a starting OD_600_ of 0.02. Ten-mL aliquots of the inoculum were dispensed into 250-mL flasks, the cultures were grown with vigorous shaking at 37 °C for 48 h, and samples were obtained every 4 h. To determine the number of viable bacterial cells at each time point, we serially diluted each sample (1:10) and 10-μL aliquots were plated on LB agar plates. The plates were incubated at 37 °C overnight and the numbers of colonies produced from each dilution was counted (colony forming units [CFU]). The final CFU/mL is obtained by the formula, CFU/mL = (number of colonies x dilution factor)/sample volume. Each experiment was performed in three independent replicates.

### Analysis of virulence factors

Different experiments were done to determine the effects of the mutation of *hepP* and *zbdP* on the expression of virulence factors as previously described. For the analysis of elastase (LasB) activity, PA14Δ*hepP* and PA14Δ*zbdP* were grown in LB broth at 37 °C; supernatants were collected at 14 h and used in the elastin Congo red tube assay [34, 37]. For pyoverdine production, the strains were grown in chelexed trypticase soy broth dialysate (TSB-DC) at 32 °C for 14 h, the supernatants were collected, and pyoverdine levels were determined spectrophotometrically at *A*_405_ [38]. The strains were grown in glycerol alanine medium at 37 °C for 24 h for evaluation of pyocyanin production [39]. Pyocyanin was extracted from the supernatant fractions with chloroform and 0.2 M HCl [37, 40]. Assays for swimming (flagellar) and twitching (pilus-associated) motilities were done as previously described [41, 42]. Strains grown overnight on LB agar plates at 37 °C were stabbed onto the surface of 1% tryptone/0.3% agar (*w*/*v*) plates (swimming motility) or to the bottom of 1% LB agar plates (twitching motility) and incubated for 16 or 24 h, respectively; zones of motility were measured as described [37, 41, 42].

The analysis of the effect *hepP* and *zbdP* mutations on the expression of quorum-sensing (QS) genes and T3SS genes was done by RT-qPCR. PA14Δ*hepP* and PA14Δ*zbdP* were grown for 16 h in LB broth for QS genes and for 16 h in TSB-DC containing nitrilotriacetic acid for T3SS genes [37] and the cells were pelleted. The RNA was extracted, purified, reverse transcribed, and subjected to qPCR as described above in Methods using primers described in Additional file 1: Table S1.

### Formation of pellicle and biofilm

The air-liquid interface (ALI) method of biofilm development was used to observe both pellicle formation and biofilm development [43–45]. The strains were grown overnight in LB broth and subcultured into fresh LB broth to a starting OD_600_ of 0.02 [41, 46, 47]. Three-mL aliquots of the inoculum were dispensed into 17 mm × 100 mm polystyrene tubes and incubated at 37 °C for 24 to 48 h. For pellicle formation, the tubes were incubated under static conditions and observed at 24 and 48 h post inoculation for pellicle formation and the relative turbidity of the broth below the ALI (planktonic growth). For the analysis of biofilm formation, the tubes were grown with gentle shaking. At 24 or 48 h post inoculation, planktonic growth was removed, the adherent biofilms were washed with distilled water to remove any nonadherent cells, and stained with 1% crystal violet. The biofilms were then washed twice with distilled water, the crystal violet eluted from the biofilms with 95% ethanol, and the color was measured spectrophotometrically at *A*_590_ [41, 46, 47].

### *Caenorhabditis elegans* slow-killing assay

This assay was performed as previously described [48]. The temperature-sensitive embryonic lethal *C. elegans* strain BW54 was obtained from the *Caenorhabditis* Genetics Center (https://cgc.umn.edu; accessed 12/12/2017) (Table 1) and maintained at 15 °C for proliferation or 25 °C for storage without proliferation. For the survival studies, 5–15 μL of overnight cultures of the test strains, PA14 as a positive control, or *E. coli* OP50, a traditional feeder source, as the negative control, were spread on 3.5-cm plates containing nematode growth agar and incubated at 37 °C for 24 h [49]. Each plate was then seeded with 10 nematodes and the plates were incubated at 25 °C to prevent proliferation of BW54. Plates were examined every day for 5 days under a dissecting microscope and the numbers of surviving worms recorded. A worm was considered dead when it stopped responding to touch. Each experiment was replicated three to six times.

### Statistical analysis

Statistical analyses of the results were done using GraphPad Prism 7.0 (GraphPad Software).

## Results

### The expression of *PA14_23420* and *PA14_23430* is influenced by thermal injury-induced changes in blood

**Fig. 1:**
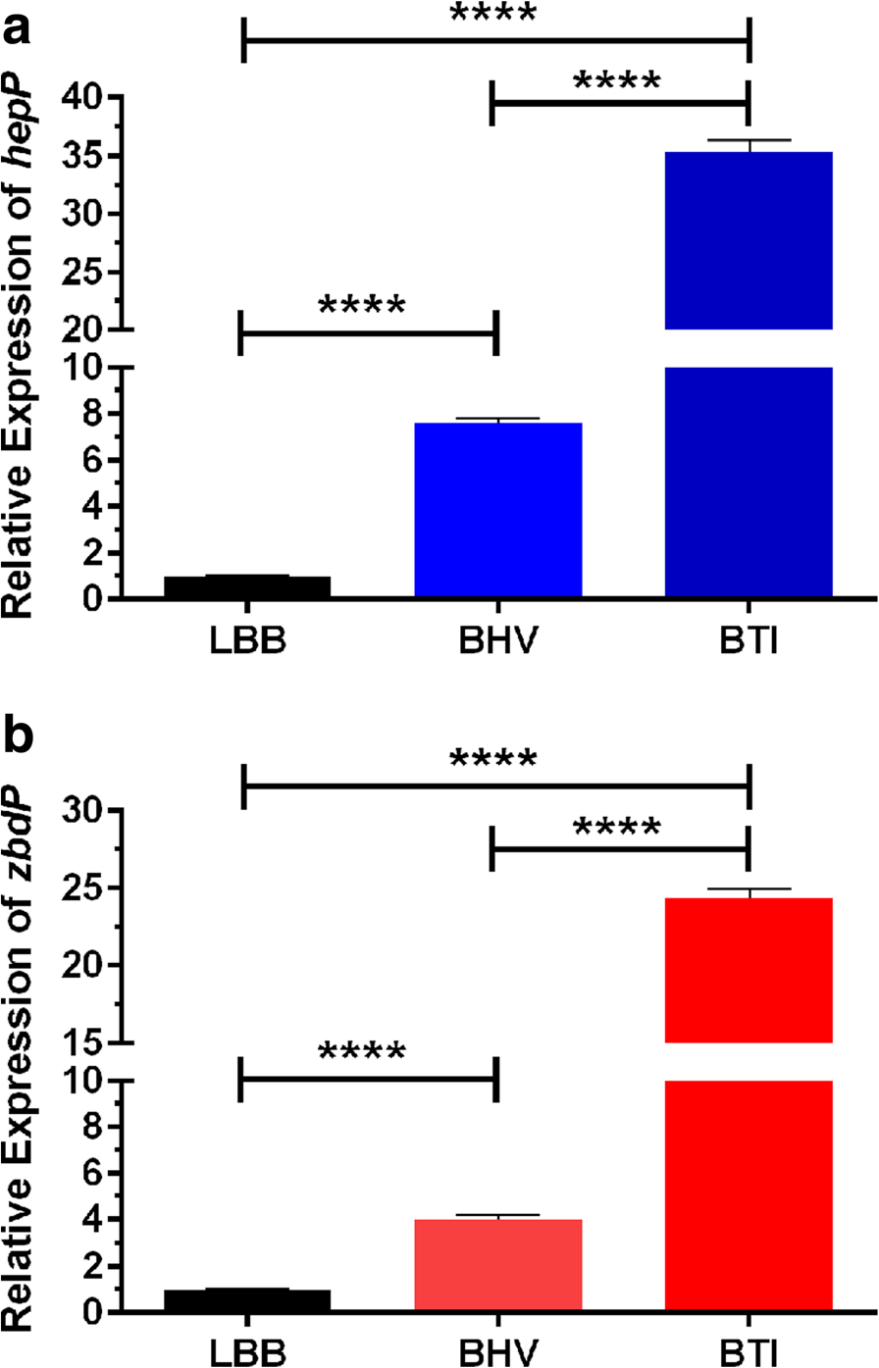
Growth of PA14 in whole blood from a severely burned patient enhanced expression of *hepP* and *zbdP*. RNA samples obtained from PA14 that was grown for 8 h at 37 °C in LB broth (LBB), whole blood from a healthy volunteer (BHV), or whole blood from a patient with severe thermal injury (BTI) were used in RT-qPCR reactions as described in Methods using primers listed in Table 2. **a** Expression of *hepP*; **b** expression of *zbdP*. Values in **a** and **b** represent the means of triplicate experiments conducted on three independently obtained samples ± SEM. ****, *P* < 0.0001

We recently examined the effect of thermal injury-induced changes in blood on the expression of *P. aeruginosa* genes [15]. *P. aeruginosa* strain PA14 was grown in whole blood from either healthy volunteers (BHV) or severely burned (thermally-injured) patients (BTI) and the level of gene expression was examined by RNA-seq analysis [15]. Among the genes whose expression was significantly enhanced were the adjacent genes *PA14_23420* and *PA14_23430. PA14_23420* (*zbdP*) encodes a hypothetical zinc binding dehydrogenase, while *PA14_23430* (*hepP*) codes for a hypothetical heparinase. Compared with BHV, the growth of PA14 in BTI enhanced the expression of *zbdP* and *hepP* by approximately seven- to eight-fold in each of three male patients (Table 3). Despite differences in age (28-62 years), percentage of total body surface involved in the burn injury (25–50%), and source of burn (fire or explosion) [15], the increase in gene expression was comparable among the three patients (Table 3). We confirmed these results by real time quantitative PCR analysis (qPCR) of the RNA samples obtained during the RNA-seq analysis. Compared with the growth in BHV, the growth of PA14 in BTI increased the expression of *hepP* and *zbdP* by approximately five- and sixfold, respectively (Fig. 1).

**Table 3:** RNA-Seq expression of *zbdP* and *hepP*

		Fold change compared to healthy volunteer^b^		
Gene	Protein Function^a^	Patient 1	Patient 2	Patient 3
*zbdP*	Zinc-binding dehydrogenase	6.8	7.5	7.9
*hepP*	Heparinase	7.1	7.6	8.1

^a^Protein functions were obtained from the *Pseudomonas Genome Database* (http://pseudomonas.com/; accessed 12/12/2017); [54]

^b^Expression of *zbdP* (*PA14_23420*) and *hepP* (*PA14_23430*) in PA14 that was grown in blood from three burn patients was compared with expression when PA14 was grown in blood from a healthy volunteer. Genes were identified as differentially expressed with ≥ two-fold change if they had the false discovery rate correction *p*-value of ≤ 0.01 [15]

In addition to the burn-induced effect, the growth of PA14 in BHV, compared with the growth in a regular laboratory medium such as LB broth, may influence the expression of either or both genes. To examine this possibility, we grew PA14 either in LB broth (LB) or in BHV and analyzed the expression of *hepP* and *zbdP* by qPCR. The growth of PA14 in BHV enhanced expression of these genes by seven- and fourfold, respectively (Fig. 1). These results suggest that, with respect to the growth in whole blood, the increase in the expression of *hepP* and *zbdP* occurs at two levels. First, there is a blood-related increase (BHV vs LB) that is likely to be triggered by certain components of whole blood; then there is a further increase (BHV vs BTI) that is likely to be triggered by thermal injury-induced changes in blood.

### *hepP* encodes a potential heparinase while *zbdP* encodes a potential zinc-binding dehydrogenase

Computer analysis of the 61.9-kDa predicted protein encoded by *hepP* using the Conserved Domains database (https://www.ncbi.nlm.nih.gov/Structure/cdd/wrpsb.cgi; accessed 12/12/2017) revealed the presence of two specific domains within the amino and carboxy terminus regions [50]. The amino-terminus domain (amino acid [aa] 116 to aa 288) has the characteristics of the chondroitin AC/alginate lyase superfamily/heparinase_II-III_N domain (Additional file 1: Figure S1a). A number of bacterial species including *P. heparinus* synthesize glycosaminoglyacans (GAGs) lyases, such as these chondroitin AC/alginate lyases, to degrade and utilize GAGs as a carbon source [24]. The carboxy terminus domain (aa 324 to aa 547) has the characteristics of the heparinase_II_III superfamily, specifically the *P. heparinus* heparinase II/III domain (Additional file 1: Figure S1a). A homology search using Protein BLAST (https://blast.ncbi.nlm.nih.gov/Blast.cgi; accessed 12/12/2017) and the nonredundant protein sequences database revealed that this domain in HepP is homologous to domains found in predicted proteins encoded by several other bacteria including *Pseudomonas fluorescens*, multiple *Acinetobacter* spp., *Shigella boydii, Edwardsiella tarda,* and *Vibrio vulnificus* (Additional file 1: Figure S1b) [51]. The strongest overall homology exists between the PA14 HepP and *P. fluorescens* F113 heparinase (Additional file 1: Figure S1b). Additional analysis for localization of HepP using the database PSORTb (http://www.psort.org/psortb/; version 3.0.2; accessed 12/12/2017) failed to detect a typical type 1 protein export signal or a cleavage site within the amino terminus of the predicted protein (data not shown) [52, 53]. Thus, whether the HepP is extracellular or cytoplasmic is unknown.

A similar computer analysis conducted on the 78-kDa predicted protein encoded by *zbdP*, revealed the presence of five conserved domains; two within the amino terminus regions and three within the carboxy terminus regions (Additional file 1: Figure S2). The amino-terminus domains are the 2-deacetyl-2-hydroxyethyl bacteriochlorophyllide-like medium chain dehydrogenase reductase (MDR) (aa 81 to aa 373) which is overlapped by the threonine dehydrogenase or related zinc-dependent dehydrogenase (Tdh) domain (aa 9 to aa 373). Additionally, the conserved putative NAD(P) binding sites found in MDR/Tdh proteins were also detected. The three conserved C-terminus domains include the NADB_Rossmann domain (aa 402 to aa 524), the oxidoreductase Gfo/Idh/MocA domain (aa 562 to aa 645), and the MviM or predicted dehydrogenase family domain (aa 402 to aa 714) which includes both of the other C-terminus domains.

**Fig. 2:**
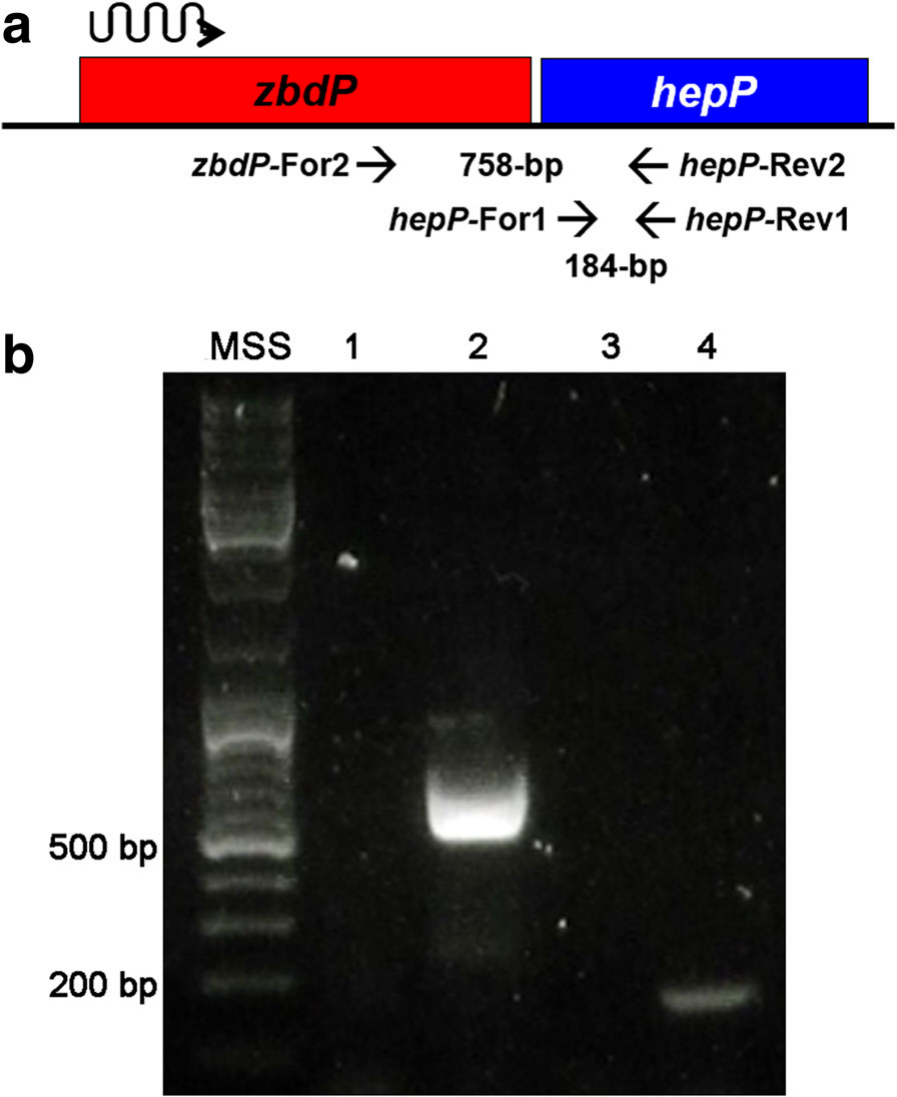
The genes *zbdP* and *hepP* constitute an operon. **a** Diagram of the location of *zbdP* and *hepP* on the PA14 chromosome. Relative size, spacing, and direction of transcription (wavy arrow) are indicated. Location of primer pairs (*zbdP*-F1/*hepP*-R1 and *hepP*-F1/*hepP*-R2) used to determine transcripts is shown by arrows. The expected sizes of the PCR products are specified on the diagram. **b** PCR products obtained from RT-PCR experiments. PA14 was grown in LB broth, pelleted, and the RNA extracted. RT-PCR was used to obtain cDNA, which served as a template in PCR reactions with the primer pairs indicated in **a**. PCR products were separated on a 0.75% agarose gel and stained with Gelstar. Lanes: 1) 1 kb DNA ladder; 2) no template control; 3) product from primer pair *zbdP*-F1/*hepP*-R1; 4) *hepP*-F1/*hepP*-R2 primer pair (positive control) (Table 2)

Computer analysis using the *Pseudomonas* Genomic Database [54] revealed that *zbdP* is located immediately upstream of *hepP* with the two genes separated by eight bp (Additional file 1: Figure S3), which suggests the two genes constitute an operon. To confirm this, RNA was extracted from PA14 grown in LB broth at 37 °C for 8 h and used in RT-PCR reactions with primers corresponding to specific sequences within *hepP* alone or within both genes (Fig. 2a). A reaction without template was included as a negative control (Fig. 2b). The expected 200-bp product was obtained with the primers for *hepP* alone (Fig. 2b). Additionally, we detected a 750-bp product using the primers that overlap both genes (Fig. 2b), indicating that *zbdP-hepP* are transcribed as an operon.

### Characterization of the putative heparinase encoded by *hepP*

**Fig. 3:**
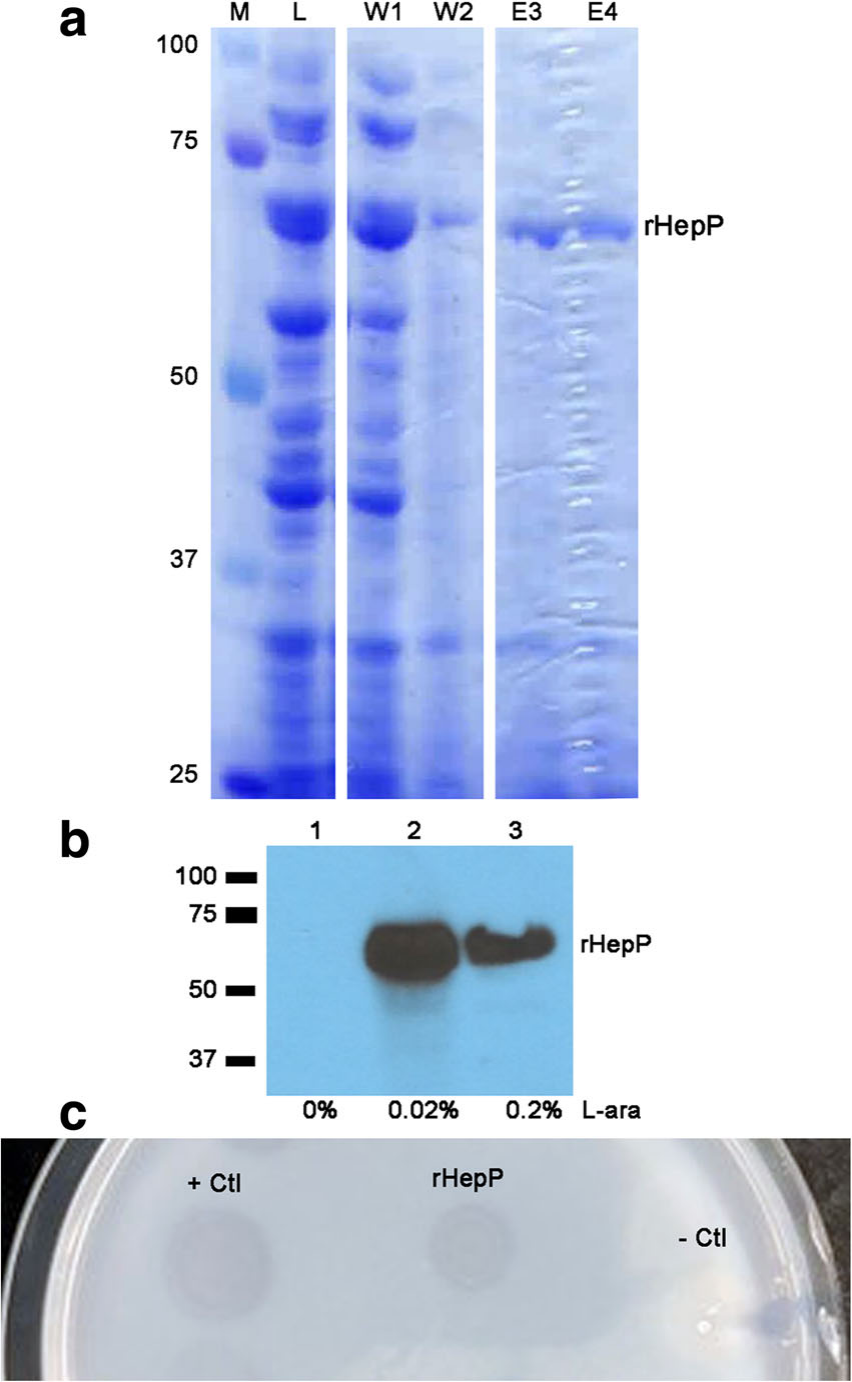
Purification and functional characterization of HepP. **a** Expression and purification of recombinant HepP (rHepP) in *E. coli*. Plasmid pND1 containing the *hepP* structural gene was expressed from the arabinose-inducible promoter pBAD. Induction experiments were conducted using 0.02% L-arabinose as described in Methods using the *E. coli* TOP10 strain as expression host. Proteins were purified by nickel column chromatography under native conditions, separated by 10% SDS PAGE and stained with Coomassie blue. Image is a compilation of lanes from the same gel. Lanes: M, molecular weight standard in kDa; L, whole cell lysate; W1 and W2, first and second of four column washes; E3 and E4, third and fourth of four elution fractions containing rHepP. **b** Immunoblotting to confirm rHepP purification. Equal amounts of purified proteins were separated by 10% SDS-PAGE, transferred to nitrocellulose membrane, and probed with anti-V5 antibody (Invitrogen). The probed membranes were treated with anti-mouse horseradish peroxidase-conjugated IgG and developed using ECL Western Blotting Substrate (Pierce). Proteins from cultures induced with 0, 0.02, and 0.2% L-arabinose (L-ara) are shown (lanes 1–3). The rHepP bands are indicated; the molecular weight standards are indicated in kDa by bars to the left of the blot. **c** rHepP exhibits heparinase activity**.** An agar plate containing 1 mg/mL porcine intestinal heparin in 1.5% agarose at pH 7 was spotted with 28 μg of rHepP in 20 μL of protein elution buffer. As a positive control (+ Ctl), 0.25 U of heparinase III from *P. heparinum* was spotted on the agar; the protein buffer was used as a negative control (− Ctl). After 1 h of incubation at 37 °C, 2% protamine sulfate solution was poured on the entire plate. Following incubation at room temperature for 2 h, the plate was examined for clear zones that indicate the presence of active heparinase

At this time, we decided to focus our efforts on *hepP* and its protein product HepP. Therefore, we obtained the 1650-bp fragment that contains the entire structural gene of *hepP* from PA14 by PCR. The gene was obtained minus the stop codon to allow synthesis of recombinant HepP (rHepP) with a 6xHis tag at the carboxy terminus. The fragment was cloned into the protein expression plasmid pBAD/Thio-TOPO, which carries the arabinose-inducible *ara*BAD promoter P_BAD_, HP-thioredoxin to maximize translation of the recombinant protein, and the 6xHis tag for purification of the protein plus the V5 epitope for recognition of the fusion protein. The resulting plasmid, pND1, was transformed into the *E. coli* expression host TOP10. Preliminary expression experiments determined that 0.02% arabinose produced optimal synthesis of rHepP (data not shown). TOP10/pND1was then grown in 50 mL of LB broth, induced with 0.02% arabinose, and cells were harvested 4 h post induction and processed as described in Methods. The rHepP was purified using nickel affinity column chromatography. Purification of rHepP was established by SDS-PAGE (Fig. 3a), and confirmed by Western blot analysis using anti-V5 antibody (Invitrogen) to detect the carboxy terminus V5 epitope within rHepP (Fig. 3b).

Once the successful purification of rHepP was confirmed, the next step was to examine its activity. To determine if the protein exhibits heparinase activity, we used the previously described heparinase plate assay [36]. An aliquot containing 28 μg of rHepP in 20 μL of protein elution buffer were spotted on the surface of the heparin-agarose plates along with the buffer as a negative control and heparinase III from *P. heparinus* as a positive control. Following incubation for 1 h at 37 °C, the plates were flooded with protamine sulfate, which reacts with intact heparin to form a white precipitate, and observed for clear zones indicative of heparinase activity. We detected clear zones with *P. heparinus* heparinase and with rHepP (Fig. 3c). These results suggest that *PA14_23430* or *hepP* does encode a heparinase enzyme.

### Examination of PA14 mutants defective in *hepP* or *zbdP*

**Fig. 4:**
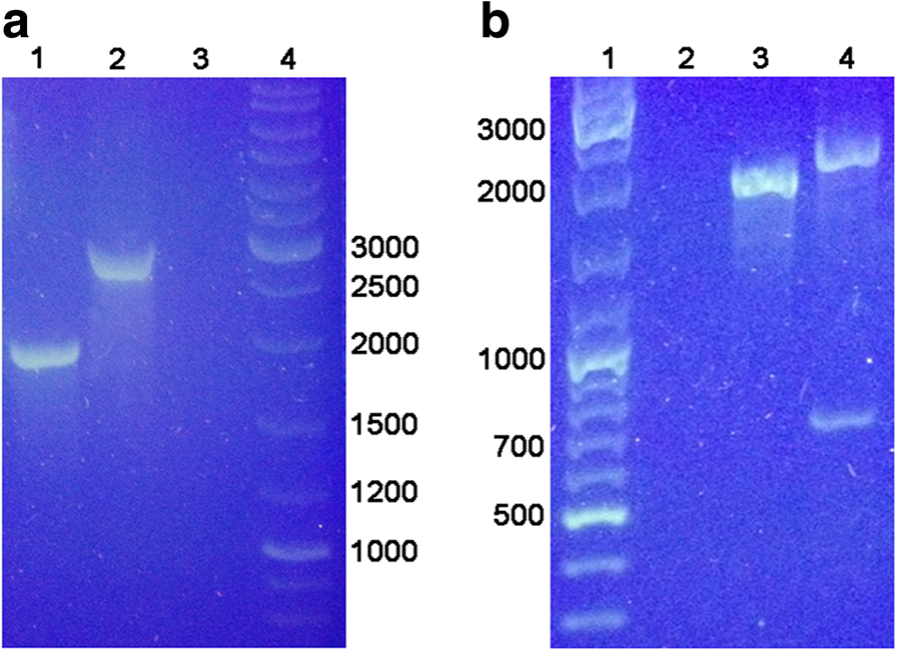
Confirmation of the mutation in PA14Δ*hepP*. PA14 and PA14Δ*hepP* were grown in LB broth and the chromosomal DNA was extracted. **a** PCR analysis to detect the presence of *MAR2xT7* within *hepP* **.** PCR reactions were run using the chromosomal DNA from each strain as a template and primers corresponding to the DNA sequences 94 bp upstream and 179 bp downstream of the *hepP* structural gene (*zbdP-*For3/*hepP-*Rev3, Table 2). The expected 1926-bp fragment from PA14 (lane 1) and the 2920-bp fragment (the additional 994 bp from *MAR2xT7*) from PA14Δ*hepP* (lane 2) were detected. Lane 3 is a no-template control and the molecular size standards are in lane 4. **b** Restriction analysis of the PCR products. The coding sequence for *hepP* does not contain an *EcoR*V restriction enzyme site, while *MAR2xT7* contains a single *EcoR*V site. Digestion of the PCR products with *EcoR*V failed to reduce the size of the 1926-bp fragment obtained from PA14 (lane 3) but resulted in the cleavage of the product obtained from PA14Δ*hepP* into the expected 800 bp and 2120 bp fragments (lane 4). Lane 1 contains the molecular size standards; lane 2 was left empty

To examine the role of HepP in the virulence of PA14, we utilized PA14 mutant strains defective in the *hepP* and *zbdP* genes. We obtained these mutants from the commercially-available non-redundant whole genome scale PA14 mutant library (PA14NR Set) [30]. These two mutants carry the Mariner transposon *MAR2xT7* [30] inserted within the structural genes; in PA14/MrT7::*PA14_23430–*480 (PA14Δ*hepP*), the transposon is inserted at nucleotide (nt) 480; while PA14/MrT7::*PA14_23420–*118 (PA14Δ*zbdP*), the transposon insertion is at nt 118. To verify the mutation in PA14Δ*hepP*, we mapped the transposon insertion using PCR and restriction enzyme analyses. PCR analysis was conducted using primers matching sequences 94-bp upstream and 179-bp downstream of the *hepP* sequence (*zbdP-*For3/*hepP-*Rev3, respectively) (Table 2, Additional file 1: Figure S3) and chromosomal DNA from PA14 and PA14Δ*hepP* as templates. We obtained the expected 1926-bp product from the chromosome of PA14 (the 1653-bp *hepP* gene plus the additional 273 nt) and the expected 2920-bp product, representing the addition of the 994-bp *MAR2xT7* transposon, from the chromosome of PA14Δ*hep* (Fig. 4a). We confirmed the presence of *MAR2xT7* within PA14Δ*zbdP* in a similar manner (data not shown).

Computer analysis revealed the presence of an *EcoR*V restriction enzyme site within *MAR2xT7* and the lack of such a site within *hepP* (http://www.restrictionmapper.org/; accessed 12/12/2017). Therefore, to further confirm the mutation of *hepP* in PA14Δ*hepP*, we performed restriction enzyme digestion on the PCR products with *EcoR*V (New England Biolabs). As expected, there was no reduction in the size of the 1926-bp from PA14 after enzyme digestion (Fig. 4b). In contrast, digestion of the PCR product from PA14Δ*hepP* produced two fragments of 800 bp and 2120 bp (Fig. 4b). The size of the two bands is consistent with the transposon insertion at 480 bp within *hepP*.

We then performed a growth curve analysis to determine if the presence of the transposon within either PA14Δ*hepP* or PA14Δ*zbdP* affected its growth. PA14, PA14Δ*hepP* and PA14Δ*zbdP* were grown in LB broth for 48 h at 37 °C. Samples were obtained every 4 h and serially diluted to determine the CFU/mL. The growth of the mutants paralleled that of their parent strain throughout the time frame of the analysis (Additional file 1: Figure S4).

### Loss of *hepP* or *zbdP* affects pellicle and biofilm formation by PA14

**Fig. 5:**
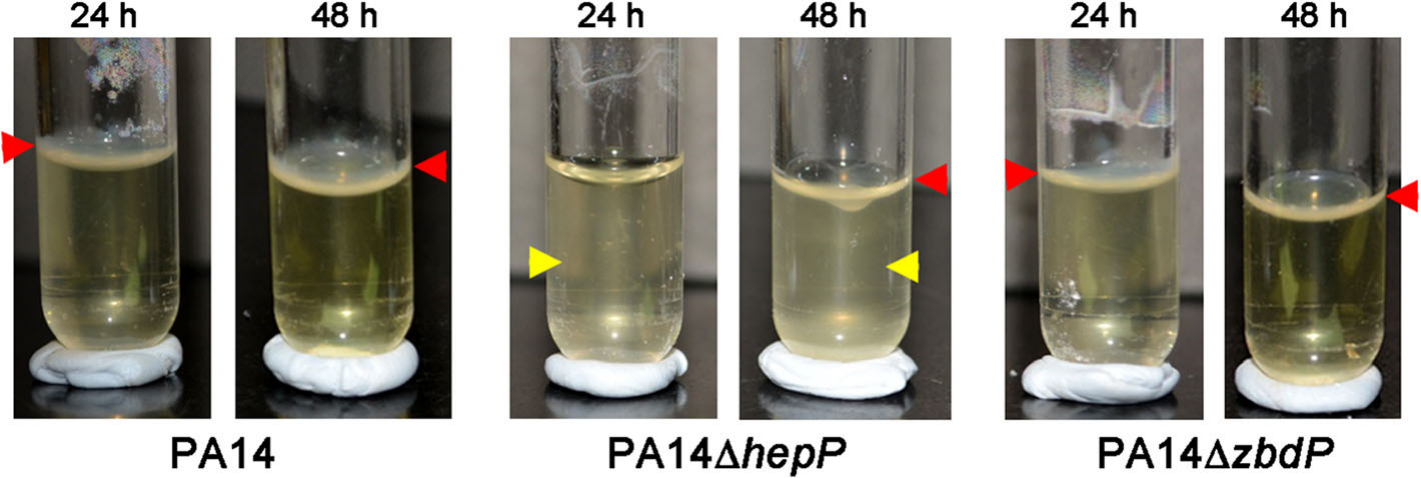
Pellicle formation is delayed in PA14Δ*hepP*. Overnight growth of PA14, PA14Δ*hepP*, and PA14Δ*zbdP* was subcultured into fresh LB broth to a starting OD_600_ of 0.02. Three-mL aliquots of the inoculum were dispensed into 17 mm × 100 mm polystyrene tubes and the tubes were incubated under static conditions at 37 °C for 24 h or 48 h. Pellicles are visible at the air-liquid interface in the tubes containing PA14 and PA14Δ*zbdP* at 24 h and with all three strains at 48 h (red arrowheads). Turbidity is present in the medium under the pellicles in the tubes containing PA14Δ*hepP* at 24 and 48 h (yellow arrowheads), while the turbidity clears from the medium in the tubes containing PA14 and PA14Δ*zbdP* at 48 h

During static growth of PA14 and PA14Δ*hepP* or PA14Δ*zbdP* in LB broth in 17 mm × 100 mm polystyrene tubes, we observed a difference between the three strains in pellicle formation. When grown under static conditions, some bacteria, including *P. aeruginosa* migrate to the air-liquid interface (ALI) and form biofilm-like structures [43]. These structures consist of bacterial cells and a pellicle, which is a matrix of extracellular polymer [45]. The ALI provides the bacteria with access to a high concentration of oxygen from the air and sufficient nutrients from the liquid. At 24 h of growth, PA14 and PA14Δ*zbdP* formed a typical pellicle while PA14Δ*hepP* did not (Fig. 5, red arrowheads). Additionally, there appeared to be more planktonic growth within the LB broth within the tube containing PA14Δ*hepP* compared to PA14 and PA14Δ*zbdP* (Fig. 5, yellow arrowhead). At 48 h, PA14Δ*hepP* appeared to form a thin pellicle, but there was still more growth within the tube compared to PA14 and PA14Δ*zbdP* (Fig. 5).

Previous studies showed that *P. aeruginosa* flagella motility contributes to pellicle formation. *P. aeruginosa* flagellum-deficient mutants either produced pellicles of unusual structure or were delayed in pellicle formation [43, 45, 55]. To assess whether the delay in pellicle formation by PA14Δ*hepP* was due to a defect in flagellum-mediated motility, we compared all three strains using the swimming and twitching assays [42]. No difference in flagellar motility or pilus-related motility was detected (data not shown).

**Fig. 6:**
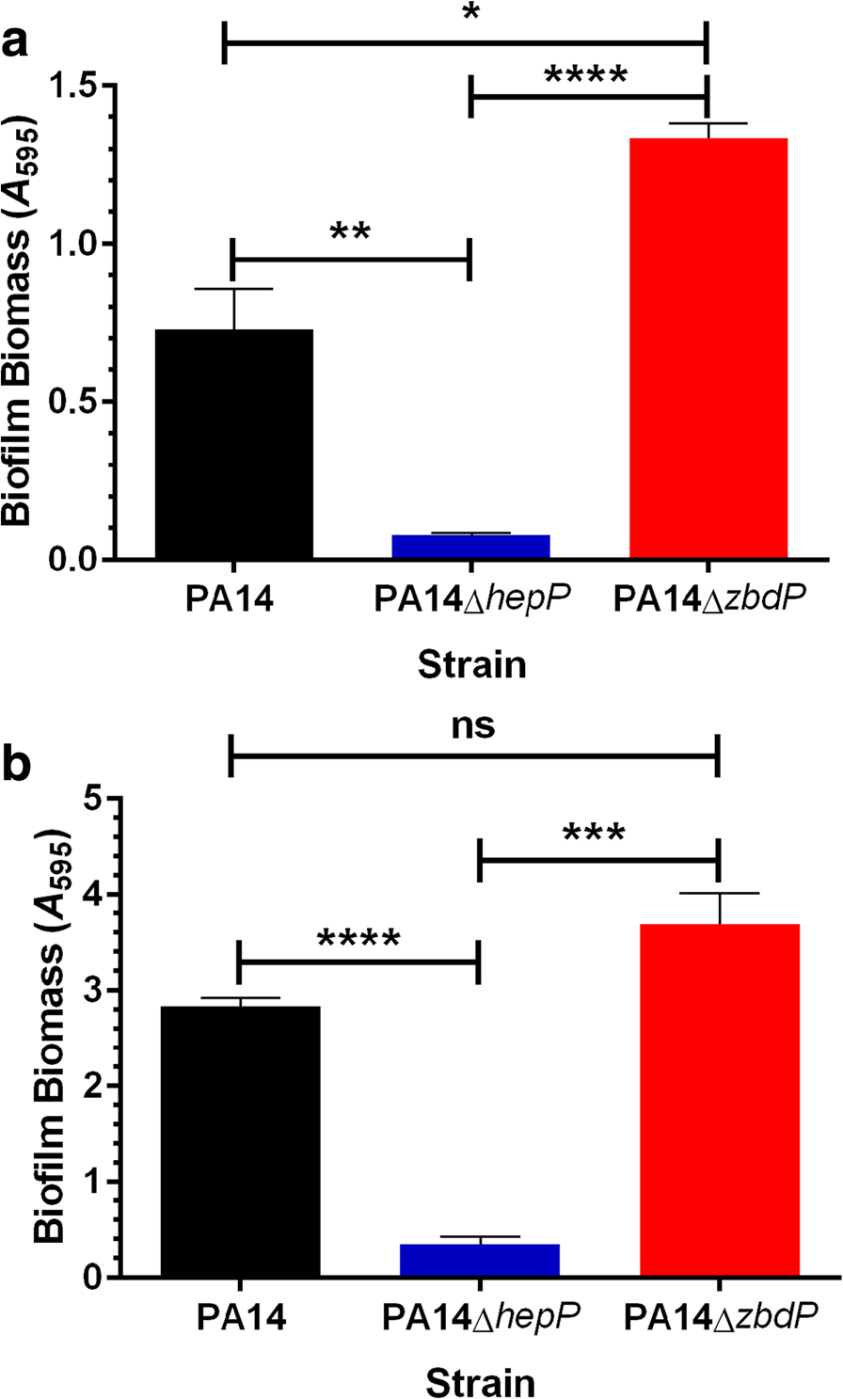
Mutation of *hepP* significantly reduced biofilm formation**.** Overnight growth of PA14, PA14Δ*hepP*, and PA14Δ*zbdP* was subcultured into fresh LB broth to a starting OD_600_ of 0.02. Three-mL aliquots of the inoculum were dispensed into 17 mm × 100 mm polystyrene tubes and the tubes were incubated at 37 °C with gentle shaking for 24 h **(a)** or 48 h **(b)**. Following incubation, the tubes were thoroughly rinsed to remove loosely attached planktonic cells and the attached cells were stained with 1% crystal violet for 1 h. The crystal violet was eluted with 95% ethanol and the eluted crystal violet, representative of the biofilm biomass, was measured at *A*_595_. Values represent means of 3 independent experiments ±SEM; *, *P* < 0.05; **, *P* < 0.01; ***, *P* < 0.001; ****, *P* < 0.0001; ns, no significant difference

Since PA14Δ*hepP* was defective in forming the biofilm-like pellicle structure at the ALI, it may also be defective in forming a biofilm on a solid surface. To examine this possibility, we compared the biofilms formed by PA14, PA14Δz*bdP*, and PA14Δ*hepP* using the ALI method of biofilm development on the walls of polystyrene tubes [42], similar to the way in which we had observed the difference in pellicle formation. All strains were grown with gentle shaking in LB broth for 24 or 48 h and the amount of biofilm formed was measured using the crystal violet assay as described in Methods [37, 46]. As shown in Fig. 6, PA14Δ*hepP* formed less biofilm than PA14 or PA14Δz*bdP* at both 24 and 48 h. Interestingly, the amount of biofilm formed by PA14Δz*bdP* was significantly higher than that formed by PA14 at 24 h (*P* < 0.05) and visibly higher (but not significantly higher) at 48 h (Fig. 6). These results suggest that the mutation in PA14Δ*hepP* affected the ability of PA14 to form biofilms on solid surfaces as well as the pellicle at the ALI, while the mutation of PA14Δz*bdP* enhanced the ability of PA14 to form biofilms on solid surfaces.

As the development of biofilms is controlled by quorum sensing (QS), we also examined the effect of the mutations in *hepP* or *zbdP* on QS-controlled virulence factors of *P. aeruginosa*. We examined the production of pyocyanin, pyoverdine and elastase (LasB) by PA14Δ*hepP* or PA14Δz*bdP* using the assays previously described [34, 38, 40, 56]. The level of each factor produced by the two mutants was comparable to that produced by PA14 (data not shown). We utilized RT-qPCR to examine the effect of the mutation on the expression of PA14 QS genes *rhlA* and *lasI*. We detected no difference in the expression of either gene (data not shown). Using the same approach, we ruled out any potential effect on the expression of the T3SS genes *exoU* and *exsD*. Again, the mutation of *hepP* did not alter the expression of either *exoU* or *exsD* (data not shown).

### Loss of *hepP* reduces PA14 virulence while loss of *zbdP* does not

The above results showed that neither *hepP* nor *zbdP* mutation affect the production or expression of several specific virulence factors. Therefore, rather than testing additional individual virulence factors, we decided to assess the effect of loss of either gene on the virulence of PA14 using the *Caenorhabditis elegans* pathogenesis model. This model, which has been used in numerous previous studies, involves the killing of the soil nematode *C. elegans* by *P. aeruginosa* [48, 57–59]. Tan et al. [48] previously demonstrated that, depending on the medium in which it is grown, PA14 produced either fast killing or slow killing of *C. elegans*. Fast killing, which occurs within a few hours, is partially mediated by bacterial secreted factors rather than live bacteria; it is produced when PA14 is grown on a high osmolarity medium. In contrast, slow killing, which occurs over the course of a few days and involves an infection process, is produced when PA14 is grown on a minimal medium (modified nematode growth media) [48].

**Fig. 7:**
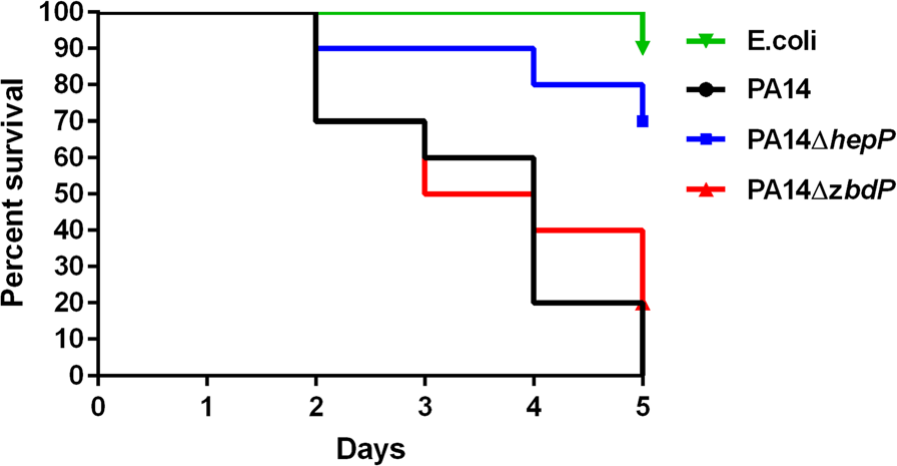
Loss of *hepP* significantly enhanced the survival of *C. elegans* infected with PA14. Adult worms were infected with wild type PA14, PA14Δ*hepP*, or PA14Δ*zbdP* and their survival was monitored daily for 5 d. Worms fed on *E. coli* OP50 were used as the control. Values represent the totals of worms used in three independent experiments. Comparison of survival curves using the Log-rank (Mantel-Cox) test produced a *P* value of <0.0001

We examined the slow killing process using *C. elegans* BW54, a temperature sensitive embryonic lethal mutant, fed on PA14, PA14Δ*hepP*, PA14Δz*bdP*, or the *E. coli* strain OP50 (the common feeder host for maintenance of *C. elegans*) as a negative control. We monitored the killing/survival of BW54 fed on the different strains for 5 d post infection. The feeding of BW54 on a lawn of OP50 produced the least lethality (90% survival) (Fig. 7). In contrast, on day 5, the survival rate of BW54 that fed on a lawn of PA14 was 0% while the survival rate of worms fed on PA14Δz*bdP* was 20% (Fig. 7). However, BW54 that fed on a lawn of PA14Δ*hepP* had a 70% survival rate on day 5 (Fig. 7). These results suggest that PA14Δ*hepP* is defective in its in vivo virulence while PA14Δz*bdP* is still virulent.

**Fig. 8:**
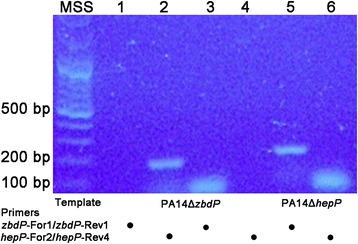
The *hepP* gene is transcribed in PA14Δ*zbdP* **.** RNA obtained from PA14Δ*zbdP*, and PA14Δ*hepP* grown in LB broth was reverse-transcribed and the cDNA used as templates in PCR reactions as indicated on the figure. Primer pairs were designed to detect internal fragments downstream of the transposon insertion in *zbdP* (*zbdP-*For1/*zbdP*-Rev1) or *hepP* (*hepP-*For2/*hepP-*Rev4). Primer pairs used in each reaction are indicated on the figure. PCR products were separated on a 0.75% agarose gel and stained with Gelstar. Lanes: MSS, molecular size standards; 1, no-template control; 2, 201-bp *hepP* product; 3, no *zbdP* product; 4, no-template control; 5, 219-bp *zbdP* product; 6, no *hepP* product

We were puzzled by the phenotypic differences between PA14Δ*hepP* and PA14Δz*bdP*. While PA14Δ*hepP* produced a significantly reduced biofilm, PA14Δz*bdP* formed a well-developed one (Fig. 6). Additionally, while PA14Δ*hepP* caused only 30% lethality among *C. elegans*, PA14Δz*bdP* caused 80% lethality (Fig. 7). The *Pseudomonas* Genomic database indicated, and our PCR analysis confirmed, that *zbdP* and *hepP* constitute an operon in which *zbdP* is the first gene and the two genes are separated by eight bp (Fig. 2 and Additional file 1: Figure S3) [54]. Our PCR analysis confirmed the presence of the operon (Fig. 2b). Therefore, we conducted RT-PCR experiments to determine if *hepP* is transcribed in PA14Δz*bdP*. RNA obtained from PA14Δ*zbdP*, and PA14Δ*hepP* grown in LB broth was used reverse-transcribed and the cDNA used as templates in experiments using primer sets designed to detect internal fragments downstream of the transposon insertion in *zbdP* (*zbdP-*For1/*zbdP*-Rev1) or *hepP* (*hepP-*For2/*hepP-*Rev4) (Additional file 1: Figure S3). As expected, analysis of RNA from PA14Δ*zbdP* showed no transcription of *zbdP* (Fig. 8, lane 3). However, we did detect the 201-bp internal *hepP* fragment from PA14Δ*zbdP* (Fig. 8, lane 2). Analysis of RNA from PA14Δ*hepP* showed the presence of the 219-bp internal *zbdP* fragment (Fig. 8, lane 5), but we detected no transcript from the region of *hepP* below the transposon insertion (Fig. 8, lane 6). These results show that although *zbdP-hepP* form an operon, loss of *zbdP* does not prevent transcription of *hepP*, which likely explains the observed phenotypic differences between the two mutants. A more detailed description of these experiments is provided in Additional file 1: Figure S5.

## Discussion

Our results showed that, compared with its growth in LB broth, the growth of PA14 in whole blood from healthy volunteers (BHV) significantly enhanced the expression of *PA14_23430*, which encodes a putative heparinase (to be called *hepP*), and *PA14_23420* that codes for a zinc-binding dehydrogenase (to be called *zbdP*) (Fig. 1). Moreover, compared with its growth in BHV, the growth of PA14 in whole blood from thermally injured patients (BTI) produced an additional increase in the expression of both genes (Fig. 1). These results suggest that these genes are related to the in vivo virulence of PA14. Previous studies have also shown that the growth of different pathogenic bacteria in blood enhanced the expression of numerous genes compared with growth in laboratory media [3, 15, 60]. For example, Graham et al. [60] showed that, compared with its growth in Todd Hewitt broth containing 0.2% yeast extract, the growth of *S. pyogenes* in blood increased the expression of 62.7% of transcripts including those of virulence factors such as *covR-covS* (*cov*, control of virulence); while Kruczek et al. [15] showed that the growth of PA14 in BTI altered (enhanced or reduced) the expression of 2596 genes compared with its growth in BHV.

Interestingly, the *hepP* and *zbdP* genes were also recovered in an earlier search for PA14 virulence-related genes done by a screening of the nonredundant PA14 transposon mutant library of 5850 clones that represent 75% of the total genome and 80% of the nonessential ORFs in this *P. aeruginosa* strain [30, 61]. Using *C. elegans* infection models and a two-step screening process, Feinbaum et al. in 2012 [61] identified 399 mutants as potentially attenuated in their virulence in the primary screening in which the virulence was measured by the reduction of the *C. elegans* brood. Of these 399 genes, 180 were identified as potentially attenuated in virulence from the secondary screening based on the slow killing of *C. elegans* [61]. Both *hepP* and *zbdP*, referred to as *ORF_11* and *ORF_10*, respectively, were identified in the primary screening; however, *zbdP* was not identified in the secondary screening suggesting that only *hepP* contributes moderately to the in vivo virulence of PA14 [61]. However, in 2006, Feinbaum and colleagues had identified both *hepP* and *zbdP* as virulence-related genes in a different study [57]. Using the same *C. elegans* slow killing assay and the same transposon mutants described (which we call PA14Δ*hepP* and (PA14Δ*zbdP*), the results of our current study support the 2012 findings, but contradict the earlier report [57, 61]. Additionally, our results suggest that *hepP* significantly contributes to PA14 virulence, rather than moderately [61], as feeding *C. elegans* PA14Δ*hepP* increased the survival of the nematodes to 70% compared to the 0% survival rate among those fed PA14 (Fig. 7).

Very little homology was discovered for *hepP* or *zbdP* at the DNA level, with only three strains of *Pseudomonas aeruginosa* (PA14OR, serotype 010, and H47921) carrying genes matching the *hepP* and *zbdP* sequences. Similarly, PCR analysis of several different *P. aeruginosa* clinical isolates previously obtained from patients at University Medical Center or the Texas Tech Physicians clinics, Lubbock, TX did not detect either *hepP* or *zbdP*. However, interrogation of the nonredundant protein database (NCBI Protein BLAST; https://blast.ncbi.nlm.nih.gov/Blast.cgi; accessed 12/12/2017) revealed that proteins with 96–100% aa identity to HepP can be found in 38 *P. aeruginosa* strains including PA14, BL04, BL16, BWH054, BWH058, BWHPSA043, BWHPSA044, CI127, HMSC057H01, and HMSC072F09; but are missing from the strains PAO1, PA103 and PAK as well as numerous other sequenced *P. aeruginosa* strains (data not shown). An additional search of the *Pseudomonas* Ortholog Groups (http://www.pseudomonas.com/orthologs/list?id=1654617; accessed 12/12/2017) found orthologous heparinases in conjunction with zinc-binding dehydrogenases in *P. aeruginosa* strains AZPAE15025 and PA14_CIA. Similarly, proteins 96–100% homologous to ZbdP were found in 31 *P. aeruginosa* including all of the strains named above except CI127. Dehydrogenase orthologs (not all were classified as zinc-binding proteins) were found in conjunction with heparinases in a number of different *P. aeruginosa* strains including 11 different AZPAE strains, WS136, Pae_CF67.11p, Pae_CF67.10q, PADK2_CF510, CF_PA39, H1I, BWHPSA008, BWHPSA010, and BHWPSA040 (data not shown).

Strains PAO1 and PA14 were originally isolated from infected wounds and both strains have been utilized extensively in in vitro and in vivo virulence studies. However, PA14 was shown to be more virulent than PAO1 in various infection models including the murine model of thermal injury and the nematode *C. elegans*, the plant *Arabidopsis thaliana*, and the wax moth *Galleria mellonella* infection models [31, 48, 62]. Using the *C. elegans* killing model, Lee et al. [57] also found PA14 to be one of the more virulent strains in their comparison of the in vivo virulence of 20 *P. aeruginosa* laboratory strains, environmental isolates and clinical isolates obtained from urine, blood, burn wounds, and respiratory secretions from cystic fibrosis (CF) patients. The CF lung isolate CF18 ranked first in virulence, PA14 ranked second, and PAO1 ranked eleventh [57]. While the PA14 and PAO1 genomes are remarkably similar, the PA14 chromosome is larger than that of PAO1 (6.5 Mb vs 6.3 Mb) [57]. Many of the PA14 genes that do not exist in PAO1 contribute to the pathogenicity of PA14 and are located within two pathogenicity islands, PAP-1 and PAP-2 [57, 63]. PAP-1 is absent from the reference strain of PAO1 and only a portion of PAP-2 is present [63]. Neither *hepP* nor *zbdP* is found within PAP-1 or PAP-2; instead they are found within a cluster of genes named PA14R38, which is present in PA14 [57]. PA14R38 was found in all 20 strains analyzed by Lee et al. [57], although the sequence of this gene cluster is so divergent that *hepP* and *zbdP* are not present in PA14R38 or any of the other 19 strains. These results suggest that *hepP* and *zbdP* genes are unique to PA14 and a number of other *P. aeruginosa* strains and were not horizontally transferred directly to them from other bacteria. However, similar heparinases can be found in the company of similar dehydrogenases in other *Pseudomonas* species, such as *P. fluorescens* F113, where the genes are in the same order and the proteins share a high level of homology (66% identity and 78% positive with no gaps for HepP; 74% identity, 85% positives with no gaps for ZbdP). The strains *P. bauzanensis* W13Z2, *P. chloritidismutans* AW-1, *P. chloroaphis* HT66, *P. denitrificans* 148-PDEN, *P. fluorescens* AU6026, AU6308, and PA4C2, *Pseudomonas* sp. PAMC 26793, and *P. stutzeri* TS44 also carry sequences encoding proteins homologous to HepP and ZbdP. These findings suggest the functions of these two proteins are conserved among many different *Pseudomonas* species*.*

The microorganism from which heparinase was fully characterized is the nonpathogenic soil microorganism *P. heparinus,* which produces three heparinases: heparinase I, which acts on heparin; heparinase II, which acts on both heparin and heparan sulfate; and heparinase III, which acts on heparan sulfate only [27]. All three *P. heparinus* heparinases are commercially available and have been used extensively in studying wound healing mechanisms such as angiogenesis [27, 64–66]. The 42.5-kDa heparinase I belongs to the alginate_lyase_2 superfamily, while the 84-kDa heparinase II belongs to the heparinase_II_III superfamily, and the 70-kDa heparinase III belongs to the alginate lyase and heparinase_II_III superfamilies.

Based on computer analysis the 61.8-kDa HepP heparinase is similar to *P. heparinus* heparinase III in that it belongs to both the alginate lyase and heparinase_II_III superfamilies. Testing of purified recombinant PA14 HepP indicated that the protein is a functional heparinase (Fig. 3c) that cleaves heparin sodium salt, which suggests that HepP functions more similarly to *P. heparinus* heparinase I or heparinase II. Whether HepP can cleave heparan sulfate is not known at this time. While the heparinase II/III domain was found within other pathogenic bacteria including *Shigella boydii, Vibrio parahemolyticus, V. vulnificus,* and *E. coli* (Additional file 1: Figure S1b), neither the genes nor their products have been characterized in any of these pathogens. Heparinase has been purified from the soil bacterium *Bacillus circulans* [67]. Although rarely found in humans, there have been a few reports of this organism causing wound infections and fatal sepsis in immunocompromised patients [68]. Despite limited aa sequence homology between *B. circulans* heparinase and *P. heparinus* heparinases, *B. circulans* heparinase degrades both heparin and heparan sulfate similar to heparinase II [69]. Unlike with *P. aeruginosa*, the virulence factors and pathogenesis of *B. circulans* have not been characterized. Regardless of its exact nature as a heparinase (I, II, or III), HepP contributes to the virulence of *P. aeruginosa* as shown by the significant decrease in mortality of *C. elegans* fed PA (Fig. 7). To our knowledge, this is the first characterized virulence-related heparinase in a bacterial pathogen.

Based on the presence of the alginate lyase domain in HepP, the protein may play a role in the cleavage of proteoglycans within the human host. Proteoglycans are composed of core proteins to which chains of anionic polysaccharides and glycosaminoglycans (GAGs) are attached. GAGs consist of repeated disaccharide units [22, 70]. Depending on the composition of these units, GAGs are classified as heparin or heparan sulfate, dermatan sulfate, chondroitin sulfate, keratin sulfate and hyaluronic acid [16, 70]. Among these classes, heparan sulfate proteoglycans (HSPGs) are the most complex and most physiologically relevant. HSPGs play a critical role in cellular processes including cell adhesion, organization of extracellular matrix, cytoskeleton organization, differentiation and morphogenesis, and tissue repair and inflammation [16, 71]. HSPGs also serve as coreceptors for cytokines, chemokines, and growth factors, a role that is associated with signal transduction; the binding of these ligands also protects them from proteolysis [71]. Shedding of the extracellular domain, leaving the membrane-spanning region with its intracellular domain intact, has been shown to play a key role in regulating the host response to tissue injury and inflammation [16, 18, 71, 72].

Many pathogenic bacteria utilize heparan sulfate for initial colonization and adherence [73]. Other pathogens, including *Enterococcus faecalis* and *Listeria monocytogenes,* utilize this mechanism for invasion and internalization [74]. The heparin-binding hemagglutinin of *Mycobacterium tuberculosis* not only facilitates adherence to epithelial cells but is also important in extrapulmonary dissemination of the infection [16, 75, 76]. *P. aeruginosa* induces shedding of the HSPG syndecan, which appears to augment its invasion of host cells [77, 78]. The shed syndecan binds proline- and arginine-rich antimicrobial peptides leading to a reduced host defense and enhanced bacterial survival and pathogenesis [71]. Despite much evidence for the importance of heparan sulfate in bacterial pathogenesis, with the exception of our current study, no previous studies have connected heparinase with bacterial virulence. One earlier study investigating the production of heparinase by *Bacteroides* concluded that heparinase produced by anaerobes is not likely to contribute to the regional thrombophlebitis that occurs during anaerobic infections [79].

Both heparan sulfate and dermatan sulfate exist in the region of the terminal web and brush border of *C. elegans* intestine [80, 81]. Furthermore, mutations in *C. elegans* genes coding for heparin, heparan sulfate, HSPG core proteins, and their biosynthetic enzymes produced several phenotypic abnormalities including defects in cytokinesis and vulval morphogenesis, developmental abnormalities at embryonic stages, abnormal axon branching, defects in the formation and maintenance of the muscle mycofilament lattice, and embryonic lethality [82, 83]. Considering our current findings, heparinase may enhance PA14 virulence in *C. elegans* by disrupting numerous physiological processes and pathways that depend on HSPGs. Therefore, the observed significant reduction in the in vivo virulence of PA14Δ*hepP* may be due to the failure of the strain to interfere with or disrupt one or more of these vital processes.

Another role for *hepP* in the virulence of PA14 is its contribution to pellicle formation at the air-liquid interface (ALI) and biofilm formation on a solid surface. With respect to pellicle formation, our results showed that when grown in a polystyrene tube under static conditions, thick pellicles are formed at the ALI by PA14 at 24 h post inoculation, but pellicle formation was delayed in PA14Δ*hepP* until 48 h, at which time a thinner pellicle became visible (Fig. 5). Previous studies showed that flagellum-mediated motility is required for pellicle formation in *P. aeruginosa* strain PAO1 [42]. A recent comparison of pellicle formation by PA14, a PA14 flagellum mutant (PA14Δ*flgk*) and a PA14 pilus mutant (PA14Δ*pilB)* also showed that loss of flagellum-mediated motility, but not pilus-mediated motility, resulted in delayed pellicle formation [84]. We ruled out the possibility that *hepP* influences flagellar- or pilus-related motility using swimming and twitching motility assays in which PA14Δ*hepP* was equal to PA14 in its motility (data not shown). Similar to the observations of Holscher et al. [84], the medium under the pellicle formed by PA14Δ*hepP* remained turbid compared to PA14 (Fig. 5). The Pel exopolysaccharide is required for the formation of pellicles at the ALI [85]. Using RT-qPCR we investigated the possibility that *hepP* influences pellicle formation through *pelC* or *pelE* (Additional file 1: Table S1). However, amount of *pelC* and *pelE* transcripts produced by PA14Δ*hepP* was comparable to that produced by PA14 (data not shown).

With respect to biofilm development on a solid surface, PA14Δ*hepP* failed to form a robust biofilm at 24 h post inoculation compared with PA14 (Fig. 6). Besides ruling out any effect of *hepP* on PA14 motility and *pel* transcription, both critical factors in biofilm initiation, we have also examined the possible effect of *hepP* on other potential contributors to biofilm development including the QS system. Assays for factors controlled by QS, such as LasB, pyocyanin, and pyoverdine, and the transcription of the QS genes *rhlA* and *lasI* produced the same results with PA14Δ*hepP* and PA14 (data not shown). Whether *hepP* affects the production of other biofilm-related factors is yet to be determined.

The genes *zbdP* and *hepP* constitute an operon (Fig. 2) whose expression in PA14 is enhanced by thermal injury-induced changes in blood (Fig. 1 and Table 3). However, it is clear that despite transposon insertion in *zbdP*, *hepP* is still transcribed in PA14Δz*bdP* (Fig. 8). Additionally, PA14 mutants defective in these genes showed phenotypic differences, with both biofilm production and lethality in the *C. elegans* model of slow killing reduced in PA14Δ*hepP* but not in PA14Δz*bdP* (Figs. 6 and 7). These results suggest that *hepP* is reinitiated from a region within *zbdP* below the transposon insertion. We are currently conducting experiments designed to identify the role of the potential zinc-binding protein encoded by *zbdP* and its relationship to the heparinase HepP.

Our work is significant because, to our knowledge, this is the first time a heparinase or heparin- and heparan sulfate-degrading enzyme has been characterized from *P. aeruginosa*. Such enzymes have been shown to have value as heparin antagonists and antineoplastic agents because of their ability to reduce neoangiogenesis [16] and heparinases I, II, and III to reduce neovascularization during tumor progression through their ability to alter the action of fibroblast growth factor [16, 20, 21, 27, 29]. In the future, heparinases may gain therapeutic value as targets for antimicrobials. We are currently conducting experiments to elucidate the effect of the HepP heparinase on PA14 virulence in a murine model.

## Conclusions

Changes within the blood of severely burned patients significantly induced expression of *hepP* in PA14. The gene codes for an active heparinase enzyme, HepP, which influences PA14 in vivo virulence. To our knowledge this is the first time a heparinase, or heparin and heparin sulfate degrading enzyme, has been characterized from *P. aeruginosa*. HepP influences pellicle formation as well as biofilm development by PA14 and the protein is required for full virulence in the *C. elegans* model of slow killing.

## Additional file

Additional file 1:
**Figure S1.** Characteristics of HepP and its homology to other proteins. **Figure S2.** Amino acid sequence of the 78-kDa predicted protein encoded by *zbdP*. **Figure S3.** Nucleotide sequences of *zbdP* and *hepP* showing locations of primers used in experiments to analyze these two genes. **Figure S4.** Mutation of *hepP* or *zbdP* does not significantly alter the growth of PA14 in vitro. **Figure S5.** Detailed experiment to confirm transcription of *hepP* in PA14Δz*bdP*. **Table S1.** Primers used to compare virulence factor gene transcription in PA14, PA14Δ*hepP* and PA14Δz*bdP.* (PDF 1972 kb)

## Abbreviations

aa: Amino acid
ALI: Air-liquid interface
BHV: Whole blood from healthy volunteers
BTI: Whole blood from severely burned (thermally-injured) patients
Cb: Carbenicillin
CFU: Colony forming units
GAGs: Glycosaminoglycans
Gm: Gentamicin
HSGAGs: Heparin and heparan sulfate glycosaminoglycans
HSPGs: Heparin sulfate proteoglycans
LB: Luria-Bertani
nt: Nucleotide
qPCR: Real time quantitative PCR
QS: Quorum sensing
RT-PCR: Reverse transcriptase PCR
Str: Streptomycin
T3SS: Type three secretion system
TSB-DC: Chelexed trypticase soy broth dialysate
