# Supplementary material for: Isolation and characterization of HepP: a virulence-related Pseudomonas aeruginosa heparinase
Source: BMC Microbiol. 2017 Dec 16;17:233. doi: 10.1186/s12866-017-1141-0 (PMC5732420; doi:10.1186/s12866-017-1141-0)
Supplement: Additional file 1: — Figure S1. Characteristics of HepP and its homology to other proteins. Figure S2. Amino acid sequence of the 78-kDa predicted protein encoded by zbdP. Figure S3. Nucleotide sequences of zbdP and hepP showing locations of primers used in experiments to analyze these two genes. Figure S4. Mutation of hepP or zbdP does not significantly alter the growth of PA14 in vitro. Figure S5. Detailed experiment to confirm transcription of hepP in PA14ΔzbdP. Table S1. Primers used to compare virulence factor gene transcription in PA14, PA14ΔhepP and PA14ΔzbdP. (PDF 1972 kb) [file 12866_2017_1141_MOESM1_ESM.pdf]

Additional file 1

This file contains:

- **Fig. A1** Characteristics of HepP and its homology to other proteins
- **Fig. A2** Amino acid sequence of the 78-kDa predicted protein encoded by *zbdP*
- **Fig. A3** Nucleotide sequences of *zbdP* and *hepP* showing locations of primers used in experiments to analyze these two genes
- **Fig. A4** Mutation of *hepP* or *zbdP* does not significantly alter the growth of PA14 *in vitro*
- **Fig. A5** Detailed experiment to confirm transcription of *hepP* in PA14Δ*zbdP*
- **Table A1** Primers used to compare virulence factor gene transcription in PA14, PA14Δ*hepP* and PA14Δ*zbdP*

a

```
  1 MALQKLVRLYHTLRYLRLKQIIFRVYYRFAQVRVHIYRNVAVRRWRRDWS  50
 51 APSWRNLSTNDAVEFTFLGITGKVQCPEDWQADRSSKLWLYNLHYLDDLN 100
101 ARDIGSQPGLADKLIQSWIQANPPVSGEGWEPYPLSLRIVNLVKWLARHD 150
151 ERSTFLADSLAVQADALVQQVEYHILGNHLFANGKALVFAGAYLSGAMAD 200
201 RWLAKGLRILDEELPEQFLNDGGHFELSPMYHATLLWDMCDLVNLSTRSG 250
251 LPDLAERLPQWREVVVQGLKWLRSMQHPDGRISFFNDAAFGIAPEYEDIA 300
301 AYAKRLDISPPAHENHLAAIYNSATGYAAVLPADGVKAILDLAKVGPDYQ 350
351 PGHAHADTLSFELSVFGKRLVVNSGTSQYGDDSERQRQRGTAAHNTVGLL 400
401 GYDSSEWAGFRVARRAAVVIERFEVEPEITCIQASHDGYGRLVKGLRHRR 450
451 TWTFTRTSMEIFDSITGDQVVATSRFFFSPEVKVSMKADGFVADMGAGKK 500
501 VSIDFSGSDDVRLISSTWHPEFGCSLANQCLVATFSGDSLTTRICWGGI  549
```

b

```
PaerPA14   324  ATGYAAVLPADGVKAILDLAKVGPDYQPGHAHADTLSFELSVFGKRLVVNSGTSQYGDdS  383
Psfluor    324  ATGYAVVRPKTGVKALLDLAQVGPDYQPGHAHADTLSFELSVFGQRLVVNSGTSQYGEGA  383
Acine_MUL  319  -SGYA-VIERNDLKLIIDVAKVGPDYQPGHAHADTLSFELSLKGRRIFVNSGTSQYGLGD  376
Enter_MUL  323  -SGYVSVV-SQNIKAILDLAKVGPDYIPGHAHADTLSFEMSFFGLRVFVNSGTSVYGLGQ  380
Sboydii    233  -SGYVSVV-SQNIKAILDLAKVGPDYIPGHAHADTLSFEMSFFGLRVFVNSGTSVYGLGQ  290
Edtarda    321  -SGYI-VINNNAYKLIIDAAKVGPDYIPGHSHADTLSFELSVEGYRVFVNSGTSLYGVSD  378
Ecoli      336  -SGYVSASTTNS-KVIMDIAKVGPDYIPGHAHADTLSYELSIFGERVIVNSGINQYGLSE  393
Vcholerae  322  SSGYSRVTFSN-YSAIFDHANVGPDYLPGHAHADTLSFELSIGKQRVFVNSGTSLYGTSA  381
Vvulnif    317  SSGYV-VVQDQANKLIIDVAKVGPDYIPGHAHADTLSFEMSIDNFRVFVNSGTSVYGLGP  375
Vparahae   300  -SGYF-IFQNQDMKSLLDLARVGPDYLPGHAHADTLSFETSFFGQRVFVNSGTSVYGEGR  357

PaerPA14   384  ERQRQRgTAAHNTVgLlGYDSSEVWAGFRVARRA---AvVIERFEVEP--EIT---CIQA  435
Psfluor    384  ERQRQRSTGAHSTVEVAGQDSSEVWAGFRVARRA---RPTIERFDLQD--GVS---YIQA  435
Acine_MUL  377  ERNRQRSTSAHNTVEVNNLNSSEVWAGFRVARRA---YPKLENIQYNNLGQVI---ALKT  428
Enter_MUL  381  ERLRQRKTQSHNTVEVDGKDSSEIWGGFRVARRA---------YPSDP--IISYDDCLTI  429
Sboydii    291  ERLRQRKTQSHNTVEVDGKDSSEIWGGFRVARRA---------YPSDP--IISYDDCLTI  339
Edtarda    379  ERLRQRKTESHNTVVVDEQDSSEVWSGFRVARRAYPTNPIIRSNN-----EVT---DIEC  430
Ecoli      394  LRLEQRKTKSHNTIEVNNMDSSEVWSGFRVAKRA---NPKVIDISLRH--ETL---LVEG  445
Vcholerae  382  ERLRQRKTPAHNTVSVSDYDSSQVWSGFRVAKRAY--AQLIKATSDEN--KVQ---LI-A  433
Vvulnif    376  ERLRQRKTNAHNTVIVDGKDSSEVWSGFRVAKRAYPSKPVIE--ESES--AIT----IEC  427
Vparahae   358  ERLRQRSTSAHNTVDVDSFNSSEVWGGFRVAKRA---IPSIPDIAVGN--NSI---SVKC  409

PaerPA14   436  S--HDGYgRLvkgLrHRRTWTFTRtSMEIFDSITGDQVVATSRFFFsPEVKV---SMkAD  490
Psfluor    436  S--HNGYRRLSSSLIHRRTWTFSEDRLEMIDQITSDQFVAVARLYIHPDVSL---SQGAS  490
Acine_MUL  429  S--HDGYVRQGVNVLHQRDWIIESNKLIVTDTLIGAYKKAQARFYLHPDVEI---KEVLE  485
Enter_MUL  430  SCGHDGYKRLPGRVDHLREWSFFDNKIIITDNLVGQFSYATAYYHLHPDIHV---EQLEN  486
Sboydii    340  SCGHDGYKRLPGRVDHLREWSFFDNKIIITDNLVGQFSYATAYYHLHPDIHV---EQLEN  396
Edtarda    431  A--HDGYVRLPGKVIHTRSWKLTHDCFIVHDNLSGDFNRASAYYHLHPNIQIDE-SIHKE  487
Ecoli      446  S--HDGYKKFSGKIIHTRKWILSDNQLIIDDFICGTFQTAISYLHFHPDISL---DVIDG  500
Vcholerae  434  R--HNGYMQQRPKVIHTRKLDCTQDSIIVSD-ILSKPVQARFHLHLHPDVNA---IKLSE  487
Vvulnif    428  S--HDGYMRLPGKVTHRRKWRLMHEQLIIHDQLSGYFNNAEAHYHVHPDIQIME-SSSDD  484
Vparahae   410  S--HNGYHRLSGKVTHVRTWSFSSSEIKIFDYLKGDFSSARVNFILHPSIRI--EKLNSQ  474

PaerPA14   491  GFVADMGAGKKVSIDFSGSDDVRLISSTWHPEFGCSLANQCL-VATFSGDSLTTRICW    547
Psfluor    491  RFVADMGTGKKVVIELAGADQVRVVASTWHPGFGCSIANQCI-EATFSGRPLTTHIHW    547
Acine_MUL  486  HSVVLILDGEEFSVSFSPNSSLDIEHSYWHPEFGKKISNYCLNIGIFDG-KLETIINW    542
Enter_MUL  487  VVQLTLSSGQKIQLESNIKIEVQ--SSTWHPEFGISVSNKKL-VIKTEG.........    532
Sboydii    397  VVQLTLSSGQKIQLESNIKIEVQ--SSTWHPEFGISVSNKKL-VIKTEG.........    442
Edtarda    488  EIIIQLPNHSQYKISAKGA-DINVSDTTWHPEFGLSVANKKI-TLKF...........    532
Ecoli      501  GVRLKLKSGKIVTLYIPCPFTVD--DSFWYPEFGVSRLNKRI-IIHLIKDQLTVTFRW    555
Vcholerae  488  KELKIIKDGEVLCLIIS-EYPITIKDSTWHPEFGKSIVNKKI-EIDFTCGLLRTEI..    540
Vvulnif    485  TVTLALPTGILYRVSVEGA-HISVVDTTWHPEFGLSIANKKL-VLNF...........    529
Vparahae   475  KYLLKLQNNKCVY--FSSNSNLFLEDYTWHPEFGKSIKTKKI-VIAMSSKKTETII..    518
```

Fig. A1 Characteristics of HepP and its homology to other proteins. a Amino acid sequence of the 61.9-kDa predicted protein encoded by *hepP*. The overlapping chondroitin AC/alginate lyase and heparinase II/III_N domains (aa 116-288) found within the amino terminus are indicated in blue letters. The heparinase II/III superfamily domain (aa 324-547) found within the carboxy terminus is indicated in red letters. The domains search was done using the Conserved Domains database (https://www.ncbi.nlm.nih.gov/Structure/cdd/wrpsb.cgi; accessed 10/29/2017)[1]. **b** Amino acid sequence homology of the HepP heparinase II/III domain

with similar predicted proteins found in other bacteria. The homology search was done using Protein BLAST (https://blast.ncbi.nlm.nih.gov/Blast.cgi; accessed 10/29/2017)[2] and the nonredundant protein sequences database. Residues within HepP that are identical to those in the majority of the other proteins are in bold red; residues in bold black indicate identity within at least one of the homologous proteins; residues in bold blue indicate similarity only was found among HepP and the homologous proteins; and lower case letters indicate no identity or similarity was present. PaerPA14, *P. aeruginosa* UCBPP-PA14; Psfluor, *P. fluorescens* F113; Acine_MUL, *Acinetobacter* multispecies; Enter_MUL, *Enterobacteriaceae* multispecies; Sboydii, *Shigella boydii*; Edtarda, *Edwardsiella tarda*; Ecoli, *Escherichia coli*; Vcholerae, *Vibrio cholerae*; Vvulnif, *V. vulnificus*; Vparahae, *V. parahaemolyticus*.

[1]Marchler-Bauer A, Bo Y, Han L, He J, Lanczycki CJ, Lu S, Chitsaz F, Derbyshire MK, Geer RC, Gonzales NR *et al*: CDD/SPARCLE: functional classification of proteins via subfamily domain architectures. *Nucleic acids research* 2017, 45(D1):D200-D203.
[2]Altschul SF, Madden TL, Schaffer AA, Zhang J, Zhang Z, Miller W, Lipman DJ. Gapped BLAST and PSI-BLAST: a new generation of protein database search programs. Nucleic Acids Res. 1997;25(17):3389-3402.

```
  1 MSFCIEGAMKQVLQDMAKGGTSVTVAPAPQISRDHVIINSTVSLISAGTE  50
 51 RMLVGFGKASYLDKARQQPEKVKMVLEKIKTDGLMTTVEAVQSKLAQPLP 100
101 LGYCNVGVVSEIGSGVEGFKVGDRVVSNGPHADLVKVSKNLCARIPDGVD 150
151 DESAAFVVVASIGLQGIRLAQPTLGECFVVTGVGLIGLLTVQLLRAHGCR 200
201 VLAIDFDDSKLELARQYGAQTCNPGKGEDPVAAGMAFSRGKGVDGVIITA 250
251 STKSSDPVTQAARMSRKRGRIVLVGVVGLELNRADFYEKELTFQVSCSYG 300
301 PGRYDPEYEEKGGDYPLGFVRWTEQRNFEAVLDMLDSGQLEVKTLITHRF 350
351 KFEDAPAAYSTLTQDNSGLGMLLLYASDTAERVVRHVPLSDMKGFNAQKP 400
401 VVGFIGAGNYASRILIPAFKTAGAQFHTLVTSGGINSVIHGEKAGFSQAS 450
451 TNIDAVLNERMINTIAIVTRHDTHARLVVDALRTGKNVFVEKPLAITHAE 500
501 LEDVQAVHAITHQTGGGPQLMVGFNRRFAPHIQKMKGLLASVREPKSFIM 550
551 TMNAGAIPVGHWTQDIAVGGGRIIGEACHFIDLMRYLAGSRIVSVQARRM 600
601 GDSNAIDTSEDKASITLGFEDGSFGTILYLANGASSFPKERIEVFTAGRV 650
651 LQLDNFRKLKGYGWPGFNKMNLWKQDKGQRQCVAAFLDSIEKGGEPTIAC 700
701 EEIFEVTRVSIEAAEILRNQWD                             722
```

Fig. A2 Amino acid sequence of the predicted 78-kDa protein encoded by *zbdP*. Analysis of the aa sequence of ZbdP using the Conserved Domains database (https://www.ncbi.nlm.nih.gov/Structure/cdd/wrpsb.cgi; accessed 10/29/2017) revealed the presence of five conserved domains.[1] Two are found in the amino terminus of the protein and three within with carboxy terminus. The 2-deacetyl-2-hydroxyethyl bacteriochlorophyllide-like medium chain dehydrogenase reductase (MDR) domain (aa 81 to aa 373) is indicated by the dashed underline. The MDR domain is overlapped by the threonine dehydrogenase/zinc-dependent dehydrogenase (Tdh) domain (aa 9 to aa 373). The additional aa within the Tdh domain are indicated by green letters. The conserved putative NAD(P)-binding sites found in MDR/Tdh proteins are indicated in bold red letters. The carboxy terminus MviM superfamily predicted dehydrogenase domain is underlined (aa 402 to aa 714). Within the MviM domain are the Rossmann-fold NAD(P)(+)-binding protein domain (aa 402 to 524) indicated by blue letters and the oxidoreductase Gfo/Idh/MocA family domain (aa 562 to 645) indicated by purple letters.

[1]Marchler-Bauer A, Bo Y, Han L, He J, Lanczycki CJ, Lu S, Chitsaz F, Derbyshire MK, Geer RC, Gonzales NR *et al*: CDD/SPARCLE: functional classification of proteins via subfamily domain architectures. *Nucleic acids research* 2017, 45(D1):D200-D203.

-500 TGCTCAAGTACTGCTCTTTTTCGTTGGTACTCTATGCTGGTACGTGCTCGTGAACGTACTCGTGCTTGTACGCAGCGTAAAATAGCAAAAAAAGGGGGGG

-400 CGATAGGGGGGCAACCGGCTATGTACGGTGCGCATGGATCTTTTATTATATTCACTCAGCTATACTTTAAGCGAGGTGCTCATGTTTTATATCCTCGCTT

-300 TTTATTTGGTGAGGAGGTTTTTGTTGCGGAGCAGTTACGATTGAATAATATGCTTATTCAGCATGTTCCTGATGTTCGGGTGTCTGATAAGGAGCATGCG

-200 TCAACCTCTAAAATTAAATTAAAATTTATTTGCCCCGAGCATAGAAAGTCATATGAGTATTTGCTGTCAAAGTTTTTTGATGATTAATTTTTTCTTTGGT

-100 TTTATATTTTGTTTGACTGCATAAGGTTTCTGTGGGACTACGTGTGTAGCAAGGATGGAGTAAATTTTTTTCTTCTTAGTTGCTATTATTTTCGTTAGGA

1 ATGAGTTTCTGTATTGAGGGCGCTATGAAGCAAGTTCTTCAAGATATGGCTAAAGGTGGCACCAGTGTCACGGTCGCTCCGGCACCGCAGATATCGCGTG

zbdP→

101 ATCATGTGATTATCAATAGTACTGTCTCGCTGATATCGGCGGGGACCGAGCGTATGTTGGTTGGCTTCGGTAAGGCATCCTACCTTGATAAGGCTCGTCA

201 ACAGCCAGAGAAGGTCAAGATGGTCTTGGAAAAGATCAAAACCGATGGATTGATGACCACAGTAGAGGCTGTACAGTCCAAGCTCGCACAGCCACTTCCC

301 CTAGGCTACTGCAATGTAGGAGTGGTGTCTGAGATCGGCTCTGGTGTTGAGGGCTTCAAGGTTGGTGATCGAGTTGTTTCCAACGGTCCTCATGCTGATC

401 TAGTCAAAGTGTCGAAGAACCTCTGTGCACGTATTCCGGATGGCGTCGATGATGAATCGGCGGCATTTGTAGTCGTGGCGAGCATTGGTCTACAGGGTAT

501 CCGTCTTGCACAACCAACCTTGGGCGAGTGTTTTGTTGTCACAGGAGTTGGCCTCATTGGTCTACTCACTGTTCAGTTGCTACGGGCTCATGGCTGT**CGG**

601 **GTGTTGGCTATTGATTT**TGATGATTCGAAGCTAGAACTTGCTCGACAGTACGGGGCGCAGACTTGTAACCCTGGTAAAGGTGAGGATCCGGTTGCTGCAG

***zbdP*-For1→**

701 GCATGGCATTCAGCCGAGGAAAAGGTGTCGACGGCGTAATTATCACTGCCTCAACAAAGTCCAGTGACCCTGTAACTCAGGCTGCACGCATGAGTC**GTAA**

801 **GCGTGGACGGATTGTT**TTGGTGGGAGTGGTGGGATTGGAGCTCAATCGCGCTGACTTTTATGAAAAGGAGTTGACTTTTCAGGTATCGTGCTCATATGGA

←*zbdP*-Rev1

901 CCGGGGCGCTATGATCCTGAATACGAGGAGAAAGGAGGAGACTACCCTCTCGGCTTTGTCCGTTGGACTGAGCAAAGAAATTTTGAAGCTGTCCTAGACA

1001 TGCTGGATAGCGGCCAACTGGAAGTGAAAACGTTGATTACTCATCGTTTTAAATTCGAGGACGCTCCGGCGGCTTATTCCACTTTAACTCAGGATAACTC

1101 TGGTCTAGGAATGCTCCTTCTGTATGCTTCGGATACGGCCGAGCGAGTTGTCCGTCATGTCCCACTATCCGATATGAAGGGCTTCAATGCTCAAAAGCCC

1201 GTGGTTGGTTTCATTGGAGCGGGAAATTACGCGTCTCGTATTCTGATCCCTGCCTTCAAGACGGCTGGAGCGCAATTCCATACTCTCGTTACCTCCGGCG

1301 GGATCAATAGTGTTATTCATGGTGAAAAAGCTGGATTCTCACAGGCATCAACCAATATTGATGCAGTGCTGAACGAGCGGATGATCAATACCATCGCTAT

1401 TGTGACCCGACACGATACCCATGCGCGTTTAGTTGTCGATGCATTGCGCACCGGGAAGAACGTTTTCGTGGAAAAGCCTCTGGCTATCACCCATGCGGAG

1501 TTGGAGGATGTACAAGCAGTCCATGCTATTACGCATCAGACCGGTGGTGGTCCGCAATTGATGGTTGGTTTCAATCGGCGTTTTGCTCCCCATATACAGA

1601 AAATGAAGGGCTTGTTGGCGTCTGTAAGGGAGCCTAAGTCCTTCATCATGACGATGAATGCAGGCGCAATTCCGGTGGGTCATTGGACTCAAGATATTGC

1701 TGTAGGTGGCGGCAGAATCATAGGTGAAGCATGCCACTTCATCGATTTGATGCGCTACCTGGCAGGAAGCCGGATAGTTTCGGTTCAGGCACGACGCATG

1801 GGAGATTCCAACGCAATTGATA**CTTCTGAGGACAAGGCTTCG**ATTACTCTAGGTTTTGAGGATGGTTCCTTCGGTACTATTCTCTACTTGGCAAACGGTG

***zbdP*-For2→**

1901 CAAGTAGTTTCCCCAAGGAGCGTATTGAGGTCTTTACTGCGGGGCGAGTTTTGCAATTGGATAATTTTAGAAAGCTGAAAGGGTATGGTTGGCCAGGTTT

2001 CAACAAGATGAATCTATGGAAGCAGGACAAAGGCCAGCGGCAGTGTGTTGCGGCGTTCCTTGATTCAATTGAGAAAGGTGGAG**AGCCGACTATCGCTTGT**

***zbdP*-For3→**

2101 **GAA**GAAATTTTCGAGGTGACGCGAGTCTCTATAGAGGCGGCAGAGATACTGCGTAATCAATGGGATTGA**GCTAGGGCATGGCACTTCAAAAACTCGTCCG**

2169 1 *hepP*→ *hepP*-ATG→

24 TCTCTATCATACGCTTCGGTACCTGAGGCTCAAGCAGATAATATTTCGAGTGTATTATCGCTTTGCCCAGGTCCGAGTTCATATTTATCGAAATGTTGCT

124 GTGCGTCGTTGGCGTAGGGACTGGTCTGCGCCCTCTTGGAGGAATTTGTCAACGAATGATGCTGTGGAGTTCACCTTTCTCGGGATAACAGGGAAAGTGC

224 AGTGCCCCGA**GGACTGGCAAGCTGATAGGA**GCAGTAAATTATGGCTCTATAATCTGCACTATTTGGATGATCTGAACGCTAGGGACATTGGCAGTCAGCC

***hepP*-For1→**

334 TGGGCTAGCCGATAAACTAATTCAAAGTTGGATCCAGGCTAATCCACCGGTATCCGGT**GAAGGTTGGGAGCCTTATCCTCTTTCCTTGCGTATC**GTTAAT

←*hepP*-Rev2 **←*hepP*-Rev1**

424 CTTGTGAAATGGCTTGCTCGCCATGATGAGCGTTCGACCTTTCTGGCGGACAGTCTAGCTGTACAAGCGGACG**CGCTGGTGCAACAAGTAGAA**TATCATA

***hepP*-For2→**

524 TCTTGGGAAATCATCTATTCGCTAATGGGAAGGCGTTGGTGTTTGCCGGCGCTTATCTGTCAGGCGCGATGGCTGATCGTTGGCTTGCCAAGGGGTTACG

624 GATACTTGACGAAGAGCTTCCTGAGCAATTTCTGAATGATGGTGGGCATTTCGAA**TTGTCTCCGATGTATCACGC**CACTTTACTCTGGGATATGTGCGAC

←*hepP*-Rev4

724 CTGGTGAATCTCTCCACGCGGTCGGGCCTACCTGATTTGGCTGAGCGTCTACCGCAATGGCGAGAGGTTGTGGTCCAAGGATTGAAGTGGCTTCGTAGCA

824 TGCAGCATCCGGATGGTAGAATTAGCTTCTTCAATGATGCTGCATTCGGAATTGCACCTGAATATGAAGATATTGCAGCGTACGCCAAGCGCCTCGATAT

924 ATCACCTCCAGCTCATGAAAACCACCTGGCGGCGATTTATAATTCGGCTACAGGTTATGCTGCTGTACTTCCTGCCGATGGCGTGAAAGCTATATTGGAT

1024 CTTGCAAAGGTCGGTCCCGACTATCAGCCGGGACATGCGCATGCCGACACATTGAGTTTTGAGTTAAGCGTCTTTGGAAAACGCTTAGTAGTCAATTCCG

1124 GAACTTCTCAGTACGGTGATGACTCTGAGCGACAAAGGCAGCGTGGTACTGCGGCGCATAATACGGTGGGATTGCTTGGCTATGATTCCTCTGAAGTTTG

1224 GGCGGGATTTCGCGTTGCAAGGCGTGCGGCAGTAGTAATTGAGCGATTCGAGGTTGAACCTGAAATAACCTGTATTCAGGCCAGCCATGATGGATATGGA

1324 AGGCTTGTGAAGGGGCTACGGCATCGTCGCACCTGGACCTTCACTCGTACGAGTATGGAAATTTTCGATAGCATAACAGGTGATCAGGTTGTGGCTACTT

1424 CTCGATTCTTCTTTAGTCCAGAAGTAAAGGTTTCTATGAAGGCCGATGGATTTGTTGCCGATATGGGGGCGGGAAAGAAAGTCTCTATCGATTTCTCGGG

1524 AAGTGATGATGTTCGTTTGATTTCTTCAACGTGGCATCCGGAGTTTGGGTGTTCATTGGCCAATCAGTGTCTCGTGGCGACTTTTAGTGGTGATTCGCTG

1624 ACT**ACTCGTATTTGCTGGGGTGGAATT**TGAAGATATTGTTGCTTAGTTTTTATTTCCATCCGGATCTGTCGGCAGGGTCGTTCCGTACTTCAGCTCTTGT

←*hepP*-[-TGA] 1653

71 GAGTGCCCTGCTTGGGGCGCTTCCAGAAAACTCGCAGTTAGAAGTTATTACTACTCAACCTAACCGTTATAGTAGTTTTTCACTTGAAG**CTCCATTGCTC**

←*hepP*-Rev3

171 **GAAAAGCAC**TCGCGACTAATTATTCATCGGATTCCTATTTTTCAGCATAATAGTGGGATGTTAGACCAGTCGCGTGCATTTTTCAGCTACCTCAAGGGCG

271 TTCTTGGTATCACTCGTAATAAGGATTATGATGTGGTATATGCAACTTCCTCTCGGTTGATGACGGCTTCTCTTGGTGCCTTGGTGGCGAGGCAATTGCG

371 ATGTCCTCTGTATTTGGATATTCGAGATATCTTCGTCGATACAATTAAGGATGTGTTGCCAAGTCGGATAAGTTTCTTTGTAAAGCCTATTTTCTCAATG

471 ATGGAGCGTATCACTGTTCGAACTGCTACC

Fig. A3 Nucleotide sequences of *zbdP* and *hepP* showing locations of primers used in experiments to analyze these two genes. The regions upstream of *zbdP* and downstream of *hepP* are indicated by gray letters. The *zbdP* gene is indicated in dark red type. Bolded, yellow-highlighted nucleotides indicate locations of *zbdP* primers used in different experiments; the names of the primers (Table 2) are indicated under each primer. The 8-bp intergenic region between *zbdP* and *hepP* is indicated by black type. The *hepP* gene is indicated in dark blue type with bold, gray-highlighted nucleotides indicating the locations of *hepP* primers. The name of each primer is given under each. Note, one primer, *hepP*-Rev3 is found in the downstream region (gray nucleotides with gray highlight). Primer pair *hepP*-ATG/*hepP*-[-TGA] was used to clone the gene for expression of recombinant HepP. The remaining primers were used in different combinations indicated within the text and/or relevant figure legends for analysis of expression of *zbdP* and *hepP*, confirmation of the transcription of *zbdP*-*hepP* as an operon, and transcription of *hepP* in PA14Δ*zbdP*. Nucelotide sequences were obtained from the *Pseudomonas Genome Database* (http://pseudomonas.com/; accessed 10/29//2017).[1]

[1]Winsor GL, Griffiths EJ, Lo R, Dhillon BK, Shay JA, Brinkman FS: Enhanced annotations and features for comparing thousands of *Pseudomonas* genomes in the *Pseudomonas* genome database. *Nucleic acids research* 2016, 44(D1):D646-D653.

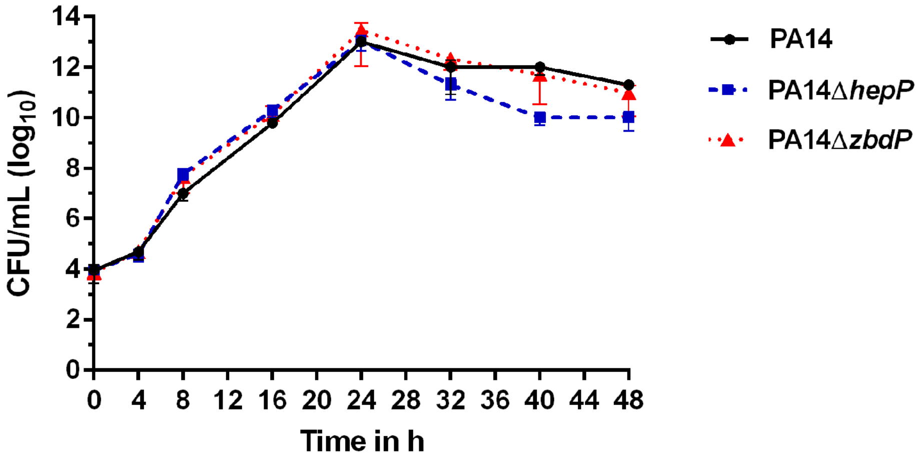

Fig. A4 Mutation of *hepP* or *zbdP* does not significantly alter the growth of PA14 *in vitro.* PA14, PA14Δ*hepP*, and PA14Δ*zbdP* were grown in LB for 48 h at 37°C. Samples were obtained at the indicated times and the CFU/mL were determined as described in Methods. Values represent the means of 3 separate experiments ± SEM.

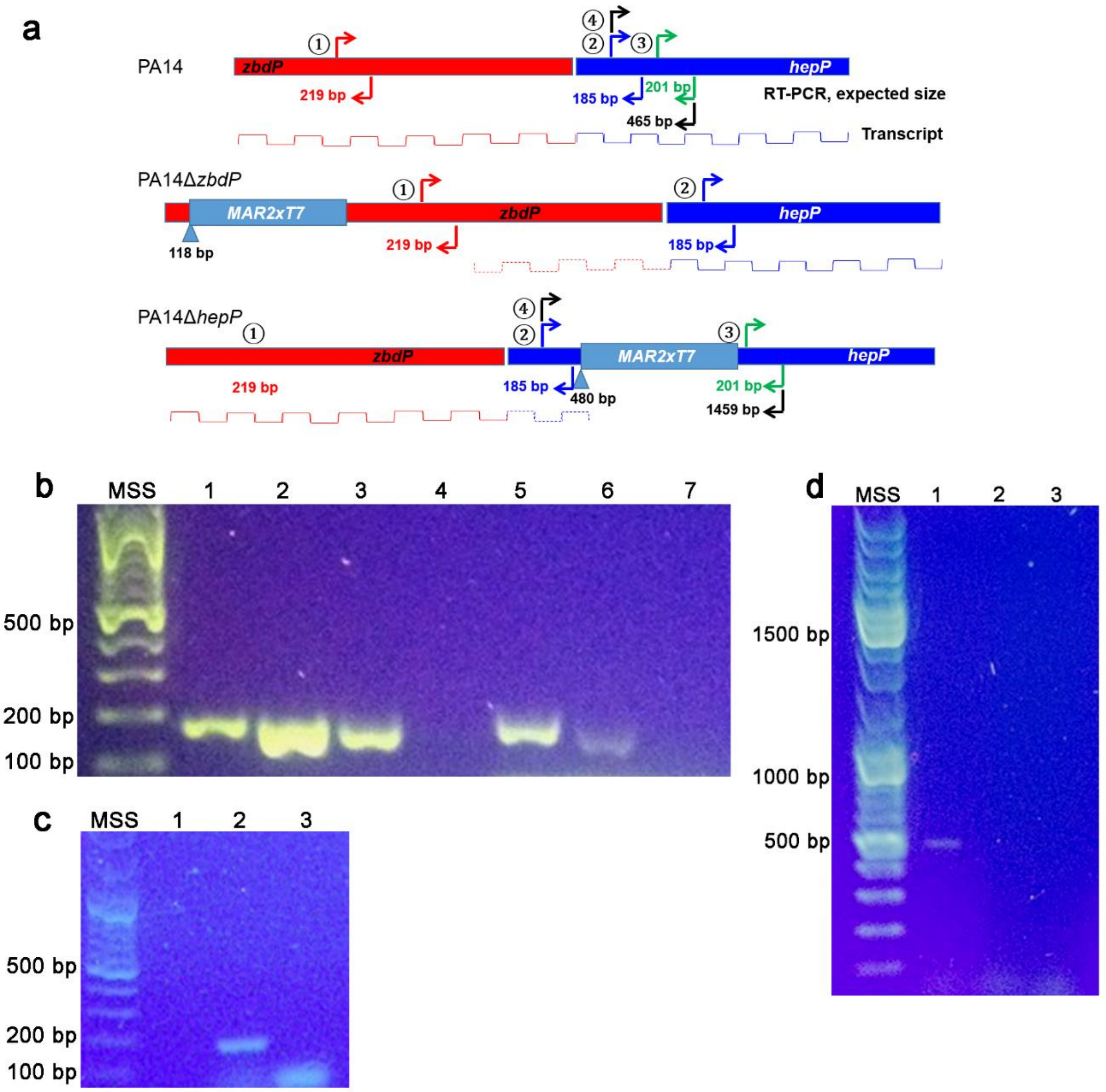

Fig. A5 Detailed experiment to confirm transcription of *hepP* in PA14Δ*zbdP*. To explore the possibility that *hepP* is being transcribed in PA14Δ*zbdP*, perhaps from an initiation site below the insertion of the transposon, we performed RT-PCR experiments on RNA isolated from PA14, PA14Δ*zbdP*, and PA14Δ*hepP*. **a Diagram of the RT-PCR experiments.** Primer pairs 1-4, their respective locations within *zbdP* and *hepP*, and the expected PCR product sizes are indicated. Transcription of the *zbdP*-*hepP* operon is indicated for PA14 and PA14Δ*hepP*. The potential reinitiation of *hepP* within PA14Δ*zbdP* is also shown. Primer pair 1, *zbdP*-For1/*zbdP*-Rev1; primer pair 2, *hepP*-For1/*hepP*-Rev1; primer pair 3, *hepP*-For2/*hepP*-Rev4; primer pair 4, *hepP*-For1/*hepP*-Rev4 (Table 2, Fig. A3). RNA obtained from PA14, PA14Δ*zbdP*, and PA14Δ*hepP* was reverse transcribed and used in PCR experiments as described in Methods. PCR products were separated on a 0.75% agarose gel and stained with Gelstar.

b *hepP* is transcribed in PA14Δ*zbdP*. PCR was done using primer pairs 1 and 2 originally used to determine whether *zbdP-hepP* were transcribed as an operon (Fig. 10a and Table 2). Lanes: MSS, molecular size standards; 1, PA14/primer pair 2; 2, PA14Δ*hepP*/primer pair 2; 3, PA14Δ*zbdP*/primer pair 2; 4, no template control/primer pair 2; 5, PA14/primer pair 1; 6, PA14Δ*hepP*/primer pair 1; 7, PA14Δ*zbdP*/primer pair 1. With primer pair 1, we found the expected 219-bp products from PA14 and PA14Δ*hepP* while no product was detected from PA14Δz*bdP* (Fig. 10b, lanes 6-8). With primer pair 2, we had expected to see a 185-bp product from PA14 and no products from the mutants (Fig. 10a). However, surprisingly, we detected the 185-bp products in both PA14Δ*hepP* and PA14Δz*bdP* (Fig. 10b, lanes 1-3), indicating that there is most likely reinitiation of transcription for *hepP* within PA14Δz*bdP*. Primer pair 2 was found to recognize a *hepP* DNA sequence upstream of the *MAR2xT7* insert, so the PCR product most likely represents the end of the transcript generated from the *zbdP* initiation site (Fig. 10a).

c *hepP* is not transcribed in PA14Δ*hepP*. We then performed a second RT-PCR experiment with primer pair 3 designed to recognize a sequence downstream of the transposon insertion (Fig. 10a). Experiments were performed as described for **b** using cDNA from PA14 and PA14Δ*hepP*. Lanes: MSS, molecular size standards; 1, no template control/primer pair 3; 2, PA14/primer pair 3; 3, PA14Δ*hepP*/primer pair 3. Now, as expected, we obtained a 201-bp product from PA14 as well as PA14Δz*bdP* (data not shown) and no product from PA14Δ*hepP* (Fig. 10c).

d *hepP* transcription is interrupted by *MAR2xT7*. As further confirmation that *hepP* is not transcribed in PA14Δ*hepP*, we did a final experiment using primer pair 4, which overlaps the region where the transposon is inserted in PA14Δ*hepP* (Fig. 10a). Experiments were performed as described for **b** using cDNA from PA14 and PA14Δ*hepP*. Lanes: MSS, molecular size standards; 1, PA14 /primer pair 4; 2, PA14Δ*hepP*/primer pair 4; 3, no template control/primer pair 4. This RT-PCR experiment returned the expected 465-bp product from PA14 while no product was detected from PA14Δ*hepP* (Fig. 10d)

These results show that although *zbdP-hepP* form an operon, loss of *zbdP* does not prevent transcription of *hepP*, which likely explains the observed phenotypic differences between the two mutants.

Table A1 Primers used for analysis of virulence factors.

Oligonucleotide	Sequence
*exoU*F	ATCACAGGTACGGCCATGTT
*exoU*R	AAGGATCAGGTTGTCGTTGC
*exsD*F	GTTACGCATCGAGCAGTTTG
*exsD*R	CTTTCCCACCAGCCATAGAC
*pelE*F	CTGTTCGAGCTGAGCAGTTG
*pelE*R	AAGAAGGCCAGGCTGAAGA
*pelC*F	GTGCCGCTGCTCAATTACTC
*pelC*R	GCCGTTCTTGTACTGCCACT
*rhlA*F	CTGAAAGCCAGCAAGCATC
*rhlA*R	GGCGGTGGTGTATTCGTC
*lasI*F	TTCCGACTGTACGCTGGAG
*lasI*R	ATCTGGGTCTTGGCATTGAG

Primers were purchased from Integrated DNA Technologies
